# Gastruloids reveal alternative morphogenetic routes for body-axis elongation with distinct cytoskeletal dependencies

**DOI:** 10.1073/pnas.2606813123

**Published:** 2026-11-06

**Authors:** Guillermo Serrano Nájera, Apolline Delahaye, Benjamin J. Steventon

**Affiliations:** ^a^ Department of Genetics, University of Cambridge, Cambridge CB2 3EH, United Kingdom; ^b^ Medical Research Council, Laboratory of Molecular Biology, Cambridge CB2 0QHUK, United Kingdom

**Keywords:** gastrulation, developmental plasticity, extracellular matrix, gastruloids

## Abstract

Morphogenesis requires cells to integrate genetic and environmental information to pattern and shape tissues. Recent studies in vivo and in vitro have shown how alterations in environmental cues can induce alternative morphogenetic programs. However, what determines which morphogenetic strategy cells deploy in different environments remains unknown. Using gastruloids, a model for mammalian posterior body-axis elongation where environmental cues can be controlled in vitro, we show that substrate availability determines which force-generation strategy cells employ to build the body axis. In free-floating conditions, gastruloids self-assemble a single posterior body axis, presumably through cell–cell interactions. When plated on laminin, gastruloids acquire a flat morphology, break symmetry multiple times, and produce several independently elongating cell streams with anterior–posterior polarity through collective cell migration. On laminin, formin activity and focal adhesion-mediated traction are required for tissue elongation, yet both are functionally dispensable in free-floating conditions. Transcriptomic analysis shows that formin inhibition blocks elongation on laminin without any detectable transcriptional changes, indicating that these cytoskeletal components play primarily a mechanical role that is only required when cells engage a substrate. Furthermore, laminin biases cells toward posterior migratory fates while preserving similar Hox expression. Together, these results show that mammalian body-axis elongation can proceed through different morphogenetic modes that engage distinct mechanical effectors depending on substrate availability. These findings have implications for understanding how evolution explores morphological diversity and how tissue engineering might achieve desired forms through precise environmental control.

## Significance

Tissues shape themselves during embryonic development through a process called morphogenesis. This requires cells to coordinate their behaviors by integrating genetic programs with environmental signals. The interplay between these factors can result in morphogenetic plasticity: variable developmental outcomes despite identical genetic backgrounds. However, how changes in the physical environment instruct morphogenetic programs, and how flexible these programs are, remains poorly understood. Here, we show that exposing gastruloids, a stem-cell model of mammalian body-axis elongation, to adherent substrates triggers a distinct morphogenetic elongation mode. Furthermore, we demonstrate that this alternative mode depends on a specific set of cytoskeletal components. This suggests developmental systems harbor latent capacities that can be unlocked by environmental context, providing potential raw material for evolutionary diversification.

Animal morphogenesis requires cells to coordinate their behaviors to build tissues and organs by integrating genetic programs with environmental signals. The interplay between these factors can result in morphogenetic plasticity: variable developmental outcomes despite identical genetic backgrounds. Recent work has demonstrated this both in vivo and in vitro, showing that geometry, mechanical properties, and chemical composition of the environment can alter morphogenetic outcomes (1–3). A clear example is gastrulation, where alterations in geometry, maternal determinants, or biochemical environment can drastically change the mode of mesoderm internalization in cnidarians, insects, and vertebrates (4). This raises a fundamental mechanistic question: when developing tissues adopt different morphogenetic strategies, to what extent does this depend on the activation of new gene-regulatory programs vs. the context-dependent use of existing cellular machinery? Understanding these interactions is critical for revealing the developmental rules underlying robustness, evolvability, and synthetic morphogenesis. However, studying them in developing embryos remains challenging due to maternal constraints, system complexity, and limited experimental accessibility. Moreover, while alternative morphogenetic outcomes have been reported in vivo and in vitro, morphogenetic plasticity itself has not been the subject of direct experimental dissection.

For these reasons, morphogenetic plasticity may be better studied in vitro, where environmental conditions can be precisely controlled. Gastruloids, stem cell aggregates that recapitulate mammalian posterior body-axis elongation (5–7), are ideal for studying how environmental context affects morphogenesis: They manifest a reproducible morphogenetic program, can be produced in high-throughput, and grown within a fully defined environment (4, 8, 9), while reproducing key aspects of mammalian gastrulation, including derivatives of the three germ layers, epithelial-to-mesenchymal transition (EMT) hallmarks, and symmetry breaking (5, 10).

However, during normal mammalian gastrulation, complex cell–substrate interactions drive key morphogenetic processes including EMT and cell migration through the primitive streak (11). This unfolds within a dynamically remodeling extracellular environment that begins with a laminin-rich basement membrane and progresses through sequential deposition of collagens and fibronectin (12). Instead, standard protocols culture gastruloids as free-floating aggregates (5), preventing substrate attachment and limiting morphogenetic forces to cell–cell interactions (13–17). Alternative approaches use micropatterning to grow embryonic stem cells on matrix-coated spots of defined size and shape, producing robust concentric germ layer patterns (18, 19). While micropatterning enables precise control over geometry and mesoderm induction sites (2), the confined nature and adhesive coatings restrict morphogenetic movements, preventing formation of a patterned body axis. As a result, how extracellular matrix (ECM) substrates affect morphogenesis in gastruloids remains untested.

Here, we show that gastruloids plated on laminin without micropatterned constraints generate posteriorly polarized cell streams using an alternative morphogenetic mode through collective cell migration. Using quantitative morphological analysis, pharmacological perturbation, and transcriptomic profiling, we demonstrate that laminin substrates induce transcriptional changes that bias cells toward posterior migratory fates while preserving the profile of Hox gene expression. On laminin, body-axis elongation requires formin activity and focal adhesion-mediated traction, yet both are functionally dispensable in free-floating conditions. Together, these results show that body-axis elongation can proceed through different morphogenetic modes that engage distinct cytoskeletal effectors depending on substrate context.

## Results

### Mesoderm Specification Regulates Gastruloid Substrate-Adhesion Profile.

Morphogenetic processes such as cell migration, epithelial folding, and tissue elongation require mechanical forces generated through cell–cell or cell–substrate interactions. In standard gastruloid culture, cells aggregate in ultrarepellent plastic wells that prevent substrate attachment (5), creating a system where morphogenesis relies mostly on cell–cell interactions to generate forces.

**Fig. 1.**
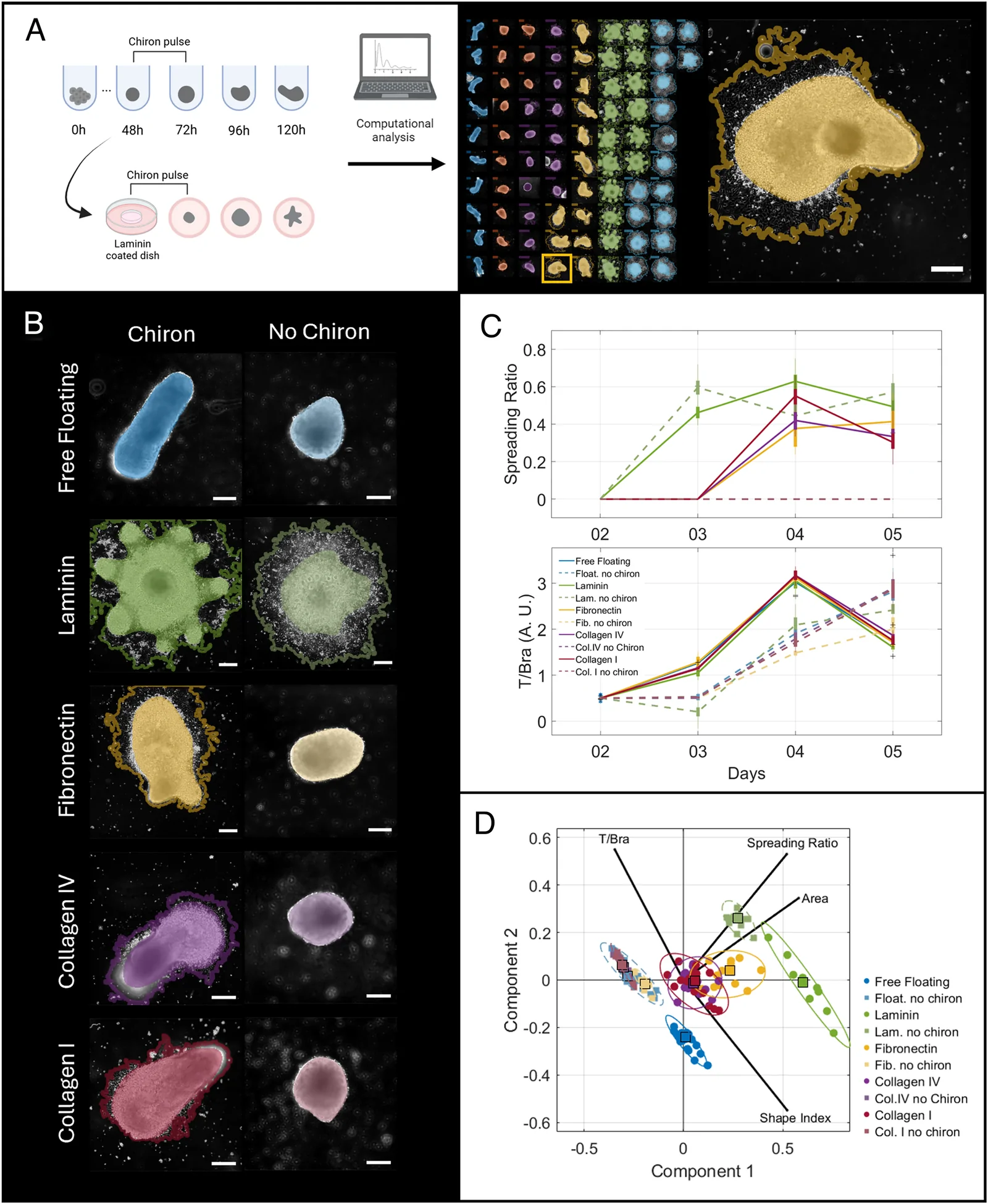
Mesoderm specification regulates gastruloid substrate-adhesion profile. (*A*) High-throughput production (*Left*) and analysis (*Center*) of gastruloids on different substrates. In the close up (*Right*) the continuous mask covers the main body of the gastruloid while the continuous line marks the limit of the migration front. (*B*) Gastruloids exhibit diverse morphogenetic behaviors depending on the substrate and the presence of Chiron. (*C*) Analysis of the spreading capability (spreading ratio) of gastruloids on different substrates (*Top*) and T/Bra expression (*Bottom*) from day 2 to 5. (*D*) Principal Component Analysis integrating different properties on day 5 (*SI Appendix*, Fig. S1).

Here, we probed the developmental potential of gastruloids in the presence of various adherent substrates. We developed an automated imaging pipeline to quantify the dynamics of reporter gene expression, morphology, and cell migration in gastruloids cultured on ECM-coated surfaces (Fig. 1*A*), with mesenchymal migration measured as the spreading ratio: the area covered by migrating cells outside the main body divided by the area of the main body (*Materials and Methods*). By day 5, gastruloids cultured on these surfaces exhibit diverse morphogenetic outcomes depending on the substrate, which differ significantly from the simple elongated shape of regular free-floating gastruloids (Fig. 1*B*). For instance, gastruloids growing on laminin develop multiple flat projections at their periphery and a halo of individually migrating cells, while gastruloids cultured on either fibronectin or collagens IV/I are characterized by a single elongating pole and variable degrees of cell migration surrounding them (Movie S1).

Interestingly, gastruloid adhesion preferences change over time (Fig. 1*C*): when exposed to ECM-coated glass surfaces after aggregation (day 2), gastruloids attach exclusively to laminin, not adhering to any other coating (fibronectin, collagen I, collagen IV); however, at later stages (day 3 to day 4), gastruloids gain the ability to adhere to all tested components, suggesting a shift in the substrate adhesion capabilities following CHIR99021 (Chiron) exposure on day 2. Since Chiron stabilizes mesoderm induction and mesoderm cells exhibit a distinct adhesion profile (20), we hypothesized that Chiron exposure could be necessary for gastruloid cells to attach to ECM proteins other than laminin. Indeed, in the absence of Chiron exposure on day 2, gastruloids failed to attach to fibronectin and collagen IV/I coated surfaces from day 3 onward, while maintaining their ability to adhere to laminin (Fig. 1 *B*–*D* and *SI Appendix*, Fig. S1). Notably, free-floating gastruloids not exposed to Chiron express T/Bra in a delayed fashion (Fig. 1*C*), but do not elongate (Fig. 1 *B*–*D* and *SI Appendix*, Fig. S1).

To evaluate whether tissue thickness influences mesoderm induction during the Chiron pulse we compared the normal protocol (where both laminin seeding and Chiron pulse occur on day 2) with two conditions that ensure the full flattening of the gastruloids prior to the addition of Chiron: early seeding (laminin seeding on day 1 and Chiron pulse on day 2), and delayed Chiron (laminin seeding on day 2, Chiron on day 3). We quantified T/Bra+ protrusion number on day 5 and found no difference in posterior tip number between the normal and early-seeding conditions, indicating that tissue thickness at the time of Chiron addition does not significantly affect mesoderm induction at this concentration and pulse duration (*SI Appendix*, Fig. S2). However, delayed Chiron addition resulted in fewer tips, demonstrating that a flat tissue cannot compensate for a postponed Chiron pulse.

Next, we tested the effects of Matrigel, a basement membrane extract mainly composed of laminin and collagen IV (21, 22). However, despite the presence of laminin, we found that gastruloids on Matrigel did not replicate the phenotype observed on laminin. Instead, Matrigel induced a morphology similar to gastruloids exposed to fibronectin and collagens (*SI Appendix*, Fig. S3*A*), with the same Chiron-dependent adhesion temporal profile (*SI Appendix*, Fig. S3*B*), which suggests the amount of laminin present in Matrigel is comparatively smaller and that other components of the extract might have a competitive effect over the laminin at this early stage.

To test laminin accessibility in relation to gastruloid flatness and stream formation, we focused on laminin and collagen IV, the two principal constituents of Matrigel. We cultured gastruloids across a range of defined laminin-to-collagen IV ratios and observed a gradual transition between the phenotypes characteristic of each pure substrate (*SI Appendix*, Fig. S4*A*). We next compared two Matrigel concentrations, 10% [within the 5 to 10% range commonly used in gastruloid studies (5, 23)] and 100%, to test whether a higher matrix concentration could recapitulate the laminin phenotype (*SI Appendix*, Fig. S4*B*). At 10%, Matrigel reproduced a phenotype resembling that of intermediate laminin-to-collagen IV ratios, whereas at 100% gastruloids remained more spherical, displaying only small patches of migrating cells at their periphery. Together, these results indicate that laminin is key in driving the strong early adhesion which could interfere with symmetry breaking.

Overall, these results demonstrate that gastruloid adhesion profiles change with their differentiation state, possibly mirroring the sequential deposition of ECM components during embryonic development. The strong affinity for laminin, independent of Chiron treatment, is consistent with laminin being the first ECM protein deposited in mammalian embryos (24), suggesting that the cellular machinery for laminin adhesion is already present before mesoderm specification.

### Gastruloids on Laminin Produce Multiple Cell Streams with Posterior Identity.

**Fig. 2.**
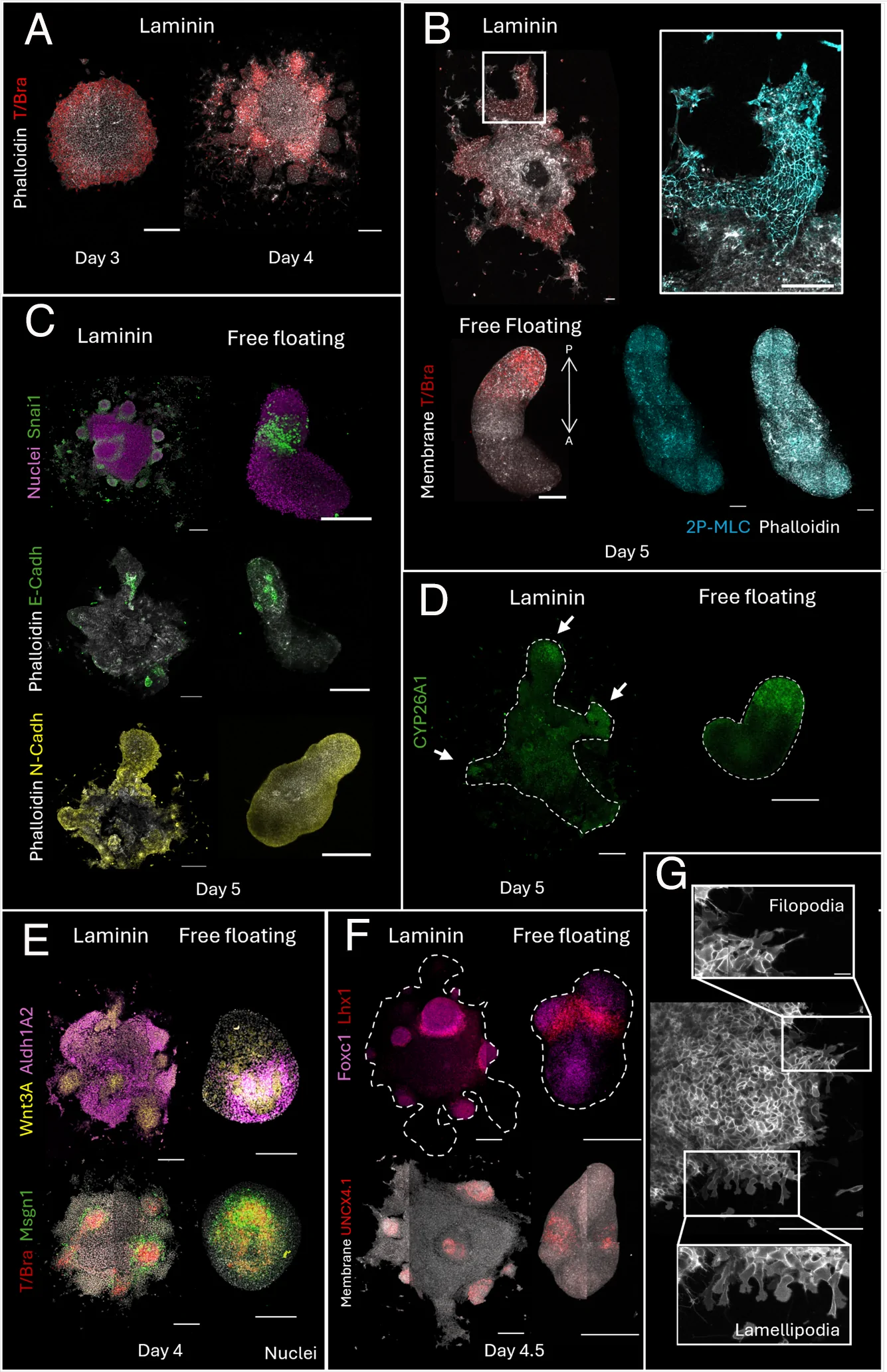
Free floating and laminin gastruloids share similar differentiation abilities. (*A*) T/Bra immunostaining of gastruloids on laminin on days 3 (n = 7) and 4 (n = 12). (*B*) T/Bra and double phosphorylated myosin immunostaining in free-floating and laminin gastruloids on day 5 (T/Bra n = 14, 30/30 protrusions; 2PMLC n = 5). (*C*) EMT-associated immunostainings on day 5 in free-floating and laminin gastruloids. (*D*) Expression of the posterior tip marker CYP26A1 in free-floating and laminin gastruloids on day 5 (n = 13 gastruloids, 26/26 protrusions). (*E*) HCR for Wnt3A, Aldh1A2, T/Bra and Msgn1 in free-floating and laminin gastruloids on day 4 (Wnt3A n = 7, 14/14 protrusions; Aldh1A2 n = 17; T/Bra, n = 21, 40/40 protrusions; Msgn1 n = 3). (*F*) HCR for Foxc1, Lhx1 and Uncx4.1 in free-floating and laminin gastruloids on day 4.5 (Foxc1 n = 5; Lhx1 n = 8; Uncx4.1 n = 7). (*G*) Confocal image of a cell stream emerging in a membrane GFP gastruloid on laminin. (Scale bars, 200 µm for the free-floating gastruloids and 500 µm for the laminin gastruloids.) n specifies the number of samples on laminin and the number of protrusions is indicated when applicable.

In free-floating gastruloids, on day 4, the emergence of the axis is preceded by the formation of a T/Bra-positive pole from an initial salt-and-pepper pattern of T/Bra-positive cells (5, 13); followed by axial elongation on day 5, driven by cell–cell interactions (15, 16, 25). We explored whether similar T/Bra-positive cell dynamics are associated with the emergence of cell streams (flat protrusions emerging from the main body between day 4 and day 5) when gastruloids are cultured on laminin. In micropatterned mouse pluripotent stem cell colonies, T/Bra-positive primitive-streak cells are first induced as a ring at the colony edge before shifting inward as outer mesoderm fates emerge (19); a comparable intermediate primitive-streak ring is seen in human colonies (18). We found that T/Bra-positive cells likewise organize as a ring at the edge of flattened gastruloids on day 3 (Fig. 2*A*). However, in contrast to confined micropatterns, where this ring is held in place and reorganizes into concentric fate domains with the mesoderm in an intermediate position, by day 4 the T/Bra cells coalesce into multiple poles that migrate outward to produce cell streams. Whereas free-floating gastruloids generated with the same cell number consolidate a single elongating axis, on laminin the cells instead form several poles (*SI Appendix*, Fig. S5), likely because adhesion to the substrate constrains the rearrangements required to merge them into one.

Next, we examined the activation of proteins associated with morphogenetic movements. We observed that the emerging cell streams on laminin are associated with a clear pattern of myosin activation (Fig. 2*B*) where double-phosphorylated (2-P) myosin is highly enriched at cellular edges within the elongating tip and completely absent in the main body of the gastruloid. Instead, in free-floating gastruloids, 2-P myosin shows a diffuse pattern without distinct domains (Fig. 2*B*). This might suggest in free-floating gastruloids multiple tissues contribute to elongation, whereas on laminin it is primarily driven by T/Bra-positive cells. However, despite these differences in its activation patterns, myosin contraction is necessary for morphogenesis in both laminin-cultured and free-floating gastruloids, as demonstrated by Rho-associated kinase (ROCK) inhibition, which hinders elongation in both scenarios (*SI Appendix*, Fig. S6). Notably, although ROCK inhibition reduced stream elongation on laminin, it increased the spreading ratio, indicating that individual cell migration persists, and even increases, in these conditions.

Beyond myosin activation, we looked at EMT markers. EMT is characterized by the downregulation of E-cad and the upregulation of N-cad and SNAI1 (26, 27). Since this cadherin switch regulates the elongation of conventional free-floating gastruloids (25), we investigated whether a similar transition occurs in gastruloids undergoing axial elongation on laminin. By day 5, SNAI1 is expressed in a band at the base of the cell streams, mirroring the pattern observed in free-floating gastruloids (Fig. 2*C*). Similar to free-floating gastruloids, E-cad expression is largely lost throughout laminin-cultured gastruloids (13, 14, 25), persisting only in patches, while N-cad is highly expressed at the outer edges of the gastruloid and within the streams (Fig. 2*C*). These results indicate that free-floating gastruloids and gastruloids on laminin undergo a comparable EMT.

We then tested whether gastruloids on laminin can generate the hallmarks of a patterned posterior body axis beyond T/Bra expression as in free-floating gastruloids. In free-floating gastruloids and embryos, the most posterior end expresses Cyp26a1, an enzyme involved in retinoic acid catabolism. Similarly, the distal ends of streams on laminin express Cyp26a1 (Fig. 2*D* and *SI Appendix*, Fig. S7), indicating their patterned posterior identity. Consistent with this, we observed the expected anterior–posterior segregation pattern: Wnt3A (posterior marker) is localized within the streams, while Aldh1A2 (anterior retinoic acid synthesis marker) is expressed in the main body of the gastruloid (Fig. 2*E*). These findings demonstrate that cell streams possess the molecular hallmarks of posterior tissues. Last, we confirmed that posterior identities are necessary for cell stream formation by shifting gastruloid tissues toward more anterior fates using Activin A, instead of Chiron (28). In these conditions, free-floating and laminin gastruloids fail to elongate, indicating that cell stream formation indeed depends on the presence of posterior cell fates (*SI Appendix*, Fig. S8).

Furthermore, we observed enhanced spatial separation of mesodermal derivatives when gastruloids are cultured on laminin. This includes distinct localization of early (Msgn1, Fig. 2*E*) and late (Foxc1, Uncx4.1, Fig. 2*F*) paraxial mesoderm markers, and intermediate mesoderm markers (Lhx1, Fig. 2*F*). Notably, gastruloids on laminin can form bulging structures that often flank the cell streams and express somite-associated markers (Foxc1, Uncx4.1, Fig. 2*F* and *SI Appendix*, Fig. S7 *D* and *E*) in organized clusters, contrasting with the more sparsely distributed expression observed in free-floating gastruloids (Fig. 2*F*). This enhanced organization supports the idea that laminin promotes somitogenesis in gastruloids, consistent with previous studies using Matrigel to induce somite-like structures (10, 23).

Finally, we performed live imaging using a line expressing EGFP at the cell membrane (GPI-GFP, see *Materials and Methods*) to capture the cellular behaviors associated with cell stream elongation (Movie S2). We observed that the emergence of cell streams is a highly dynamic process, with cells migrating on the substrate while extending active filopodia and lamellipodia (Fig. 2*G*), actin-based subcellular structures with important roles in cell movement. Altogether, these results suggest that cell streams represent posteriorly polarized structures that elongate using different cell behaviors and myosin activation patterns than their free-floating counterparts.

### Inhibition of Lamellipodia Formation Enhances Collective Cell Migration on Laminin.

**Fig. 3.**
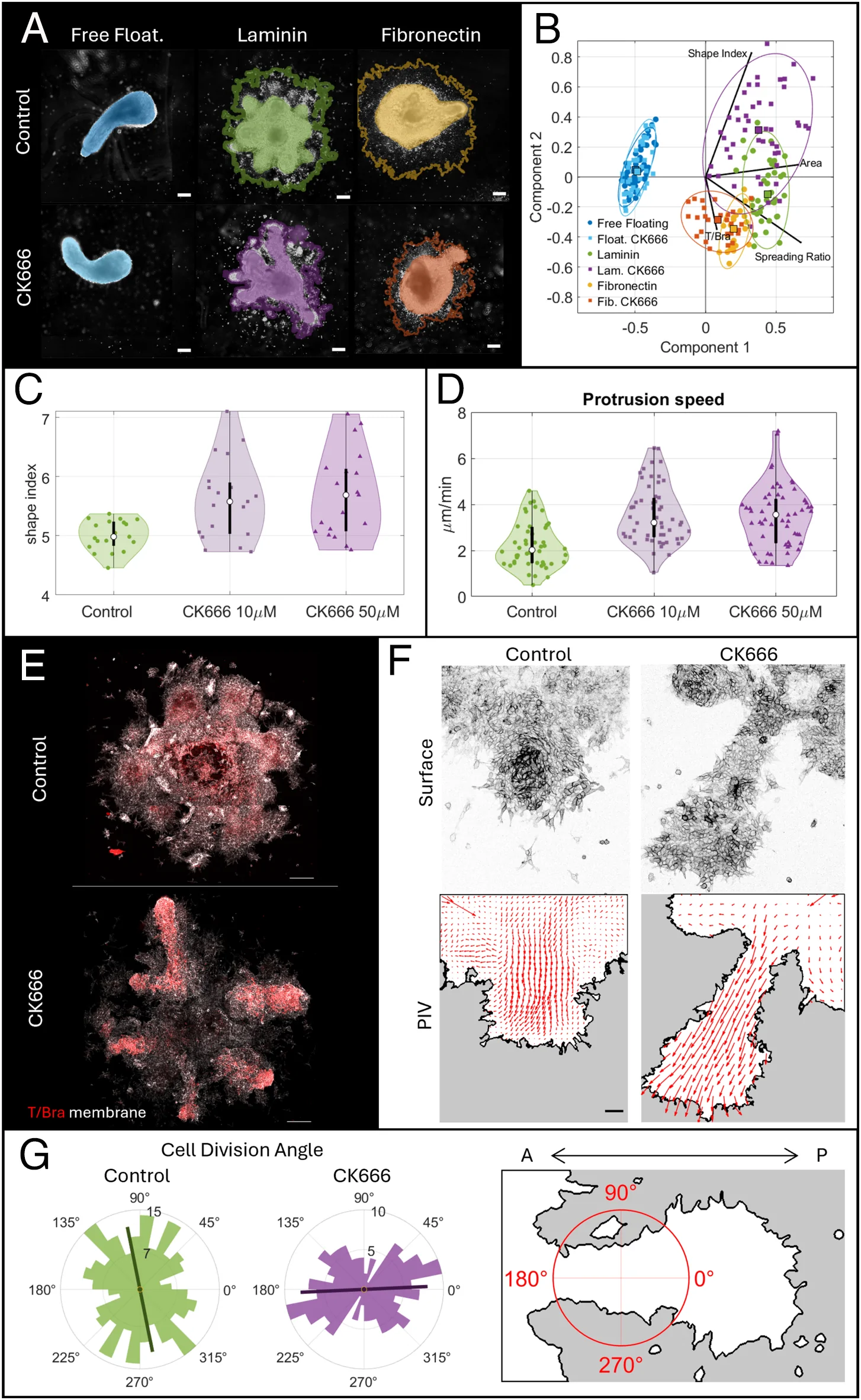
Inhibition of lamellipodia enhances elongation on laminin-seeded but not on free-floating gastruloids. (*A*) Gastruloid morphologies on different substrates with or without CK666 50 µM at day 5. (*B*) Principal component analysis integrating different properties on day 5 (*SI Appendix*, Fig. S10*A*). (*C*) Quantification of the shape-index with different CK666 concentrations. (*D*) Quantification of the migration speed of T/Bra positive tips. (*E*) High-resolution image demonstrating the higher elongation capabilities of laminin-seeded gastruloids treated with CK666. (*F*) Particle image velocimetry on the developing cell streams (*SI Appendix*, Fig. S11). (*G*) Quantification of the cell division angle with respect to axis of elongation. See Dataset S1 for full statistical analysis. (Scale bars, 200 µm in *A*–*E* and 50 µm in *F*. Gastruloids treated with CK666 at day 4 [96 h]).

Lamellipodia are dense, two-dimensional branched networks of actin filaments important for cell locomotion. To assess the role of lamellipodia in the elongation of cell streams on laminin, we used CK666 (Fig. 3), an inhibitor of the Arp2/3 complex (29, 30). This complex is an actin nucleator that organizes actin fibers in a branching configuration necessary for lamellipodia formation (31, 32). As expected, adding CK666 at day 4 reduced the formation of lamellipodia (*SI Appendix*, Fig. S9) and the individual cell migration on both laminin and fibronectin (Fig. 3 *A* and *B* and *SI Appendix*, Fig. S10*A*), suggesting that lamellipodia are required for the effective directed displacement of individual cells.

However, surprisingly, CK666 treatment resulted in significantly longer cell streams protruding from gastruloids on laminin (Fig. 3 *C* and *E*), with no measurable effect on the elongation of free-floating gastruloids or those seeded on fibronectin (*SI Appendix*, Fig. S10). To quantify these differences, we decomposed gastruloid morphology using LOCO-EFA modes (*SI Appendix*, Fig. S10), a series of unbiased bidimensional shape descriptors based on Fourier transforms (33). While the first two modes describe ellipsoidal shapes, higher frequencies characterize more complex morphologies. This analysis confirmed that CK666 shifted laminin-plated gastruloids toward more complex, elongated shapes without affecting free-floating gastruloids or those on fibronectin (*SI Appendix*, Fig. S10*C*).

Next, we sought to identify the underlying cause of the more elongated morphology. Since there was no change in the area of laminin-plated gastruloids after CK666 treatment (*SI Appendix*, Fig. S10), we hypothesized that the elongation resulted from enhanced migration capabilities of cell streams (Movie S3). To test this, we quantified the migration speed of cell stream tips from gastruloids on laminin exposed to CK666 (Movie S4). Our measurements revealed that migration velocity increased, confirming that inhibition of the Arp2/3 complex enhanced their migratory capacity (Fig. 3*D*).

To further investigate the tissue movements, we recorded high-resolution time-lapse movies of protrusion formation using the GPI-GFP line with or without CK666. Particle image velocimetry (PIV) analysis of these movies suggested that cells move in a coherent directional manner with an increased velocity in the presence of CK666 (Fig. 3*F* and *SI Appendix*, Fig. S11). In both conditions, we did not observe significant oriented cell intercalations once cells abandoned the main gastruloid body and entered the stream, which suggests that the formation of these streams represents a form of collective cell migration rather than convergent-extension fueled by oriented cell intercalations. The pattern of tissue flows was not affected by the addition of CK666, except for the enhanced migration speed.

Finally, we observed that cell divisions in the stalk connecting the most distal (posterior) tip with the main gastruloid body preferentially orient perpendicular to the elongation axis (Fig. 3*G*). We hypothesized that lamellipodia at the perimeter of the stream (Fig. 2*G*) generate lateral pulling forces that bias cell division angles (Fig. 3*G*). These perpendicular forces are presumably counterproductive for the cell stream formation since they contribute to lateral rather than axial elongation. To address this idea we followed the separation of daughter cells in the elongation region in control (Movie S5) and CK666-treated (Movie S6) gastruloids when on laminin. Strikingly, CK666 treatment caused cell division orientation to shift and statistically align with the elongation axis (Fig. 3*G*), suggesting that removing lamellipodia reduces lateral pulling, leaving only productive forces that promote axial elongation, increasing migration speed. On the whole, these experiments show that Arp2/3 has a context-dependent mechanical function: on laminin, lamellipodia may generate lateral forces that slow down axial elongation, whereas in free-floating conditions Arp2/3 inhibition has no effect.

### Cell Stream Formation on Laminin Requires Formin Activity.

**Fig. 4.**
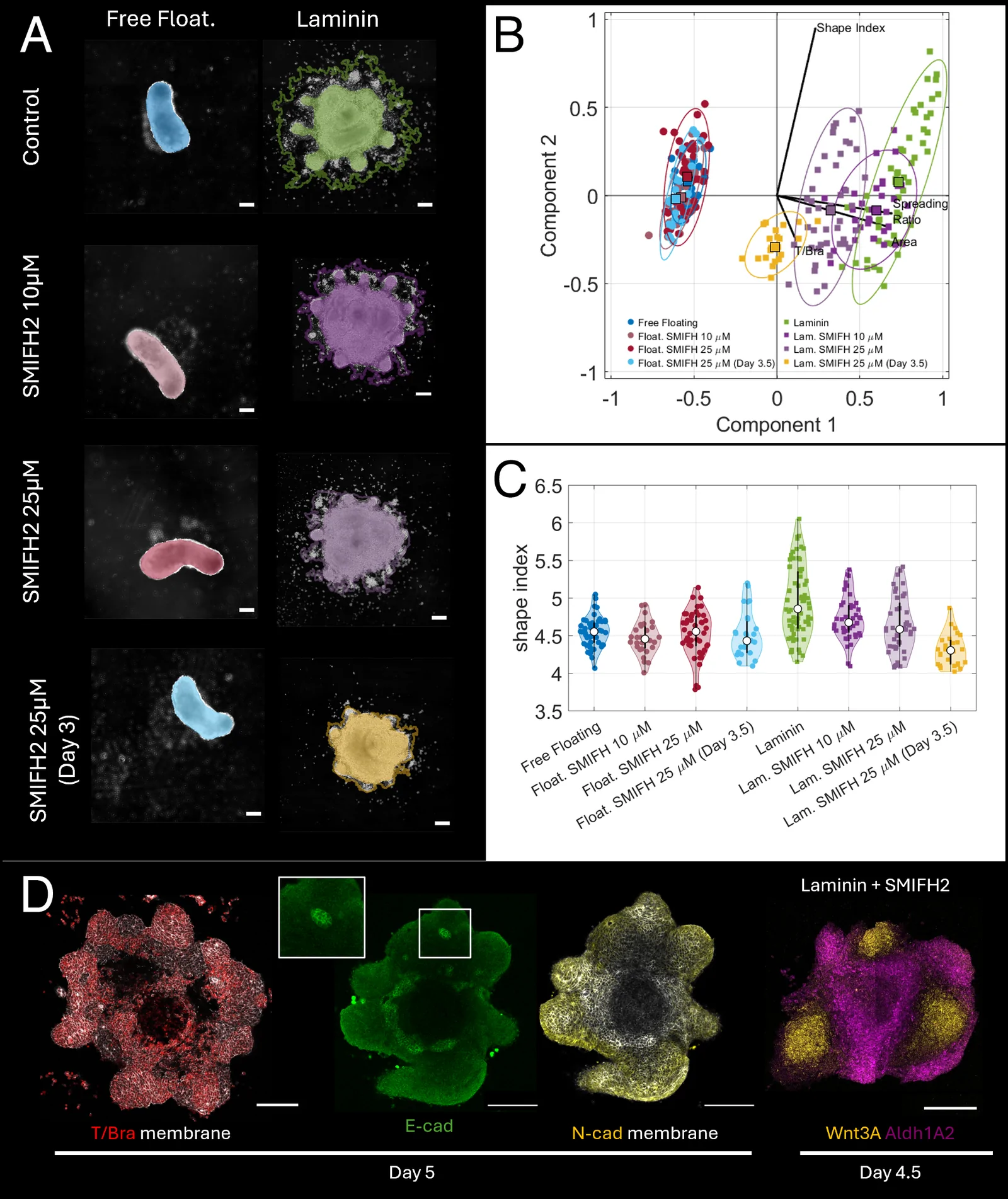
Inhibition of formin activity blocks elongation on laminin-seeded but not on free-floating gastruloids. (*A*) Gastruloid morphologies on different substrates with or without SMIFH2 at day 5. (*B*) Principal component analysis integrating different properties on day 5 (*SI Appendix*, Fig. S13*A*). (*C*) Quantification of the shape-index with different SMIFH2 concentrations. (*D*) Expression of T/Bra (reporter line), E/N-cad (immunofluorescence), and Wnt3A/Aldh1A2 (HCR) on laminin gastruloids treated with SMIFH2 25 µM at day 3 (84 h). The inset shows a patch of E-Cad expression on the cell membrane. See Dataset S1 for full statistical analysis. (Scale bars, 200 µm).

Having established that inhibiting lamellipodia formation enhances collective cell migration and stream elongation specifically on laminin, we next investigated the role of filopodia in this process. Filopodia are finger-like cellular protrusions composed of parallel bundles of actin filaments (34), which are primarily nucleated by formins (35). To determine whether these actin-rich protrusions are required for the laminin-specific cell stream elongation (Fig. 4), we treated gastruloids with SMIFH2, a small molecule inhibitor of formin homology 2 domains (FH2) (36), and several myosins (37), including myosin 10 which localizes to filopodia tips. We therefore interpret SMIFH2 effects as reflecting the combined inhibition of filopodia formation and formin/myosin-dependent actin structures (*Discussion*). Accordingly, we observed a reduction in filopodial length on both laminin-plated (*SI Appendix*, Fig. S9) and free-floating gastruloids across tissue depths (*SI Appendix*, Fig. S12).

Strikingly, SMIFH2 significantly blocked the formation of cell streams with its severity increasing with dose or exposure time, on gastruloids cultured on laminin, while having no effect on their free-floating counterparts (Fig. 4). The inhibitor prevented area expansion, individual cell migration, and cell stream formation in gastruloids cultured on laminin (*SI Appendix*, Fig. S13*A*), but had no measurable effect on free-floating gastruloids as shown in the integrated Principal component analysis (PCA) (Fig. 4*B*). Indeed, LOCO-EFA shape analysis demonstrated that SMIFH2 decreased the morphological complexity of laminin-plated gastruloids in a dose-dependent manner (*SI Appendix*, Fig. S13*B*). While control gastruloids on laminin developed complex shapes characterized by higher-order LOCO-EFA modes, SMIFH2-treated gastruloids remained predominantly circular (*SI Appendix*, Fig. S13*C*).

We did not observe significant changes in mesodermal differentiation (T/Bra), the cadherin switch (E-cad/N-cad), or the specification of anteroposterior markers (Aldh1A2/Wnt3A) upon addition of SMIFH2 at day 3.5 (84 h), even at the highest tested concentration (25 µM, Fig. 4*D* and *SI Appendix*, Fig. S13*D*). This suggests that filopodia inhibition does not substantially affect signaling pathways, but is instead required for substrate-engaged elongation, consistent with a role in generating force on a substrate. Moreover, if filopodia were significantly involved in signaling, we would expect to see similar effects in free-floating gastruloids, which also exhibit filopodial protrusions (*SI Appendix*, Fig. S12). Altogether, these results demonstrate that tissue elongation on laminin requires specific cytoskeletal machinery (Movie S7).

### Formin Inhibition Blocks Elongation on Laminin without Detectable Transcriptional Changes.

**Fig. 5.**
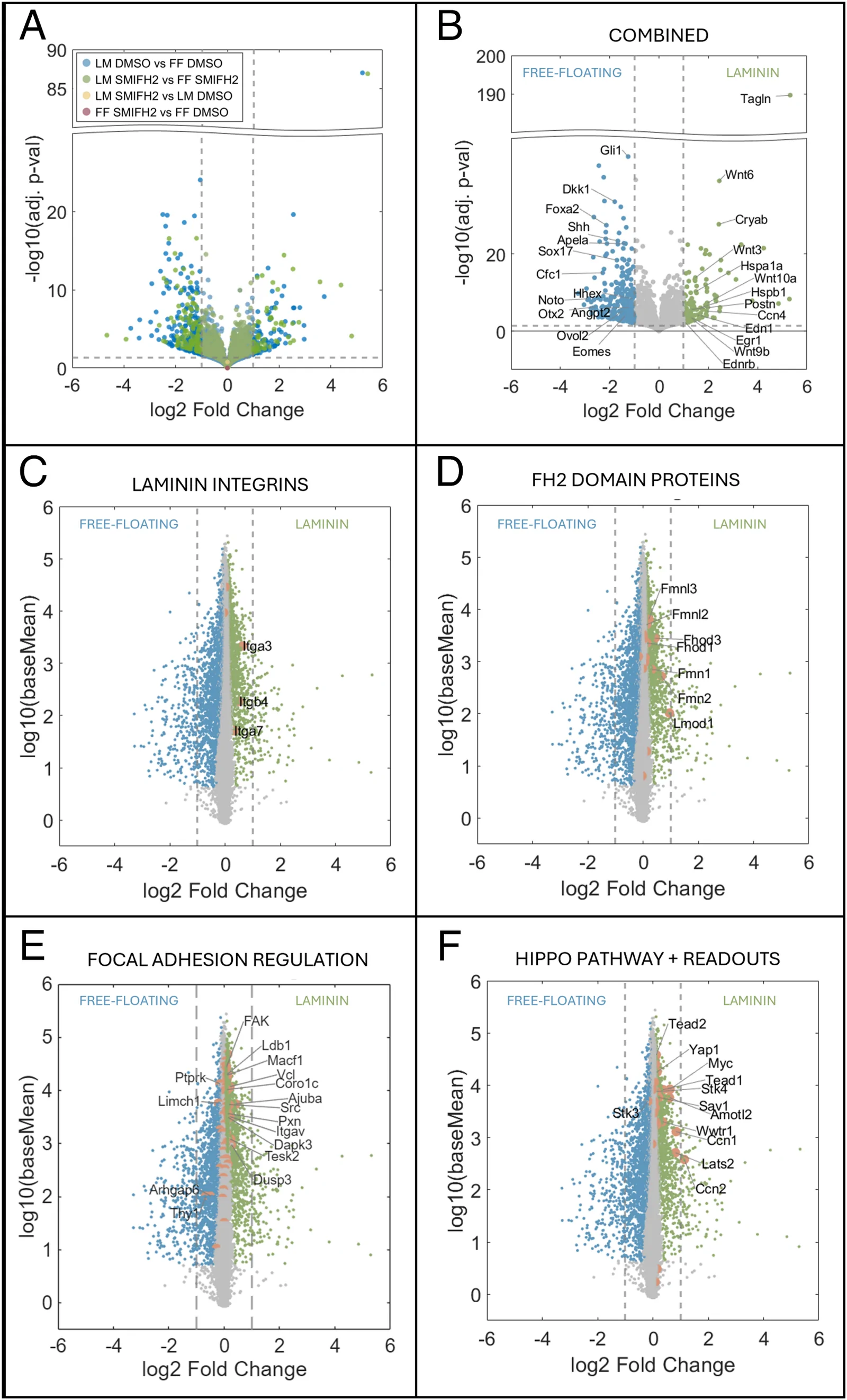
Laminin promotes the expression of genes involved in cell migration. (*A*) Formin inhibition does not induce changes in gene expression. Volcano plot showing differentially expressed genes across conditions. Blue: laminin DMSO vs. free-floating DMSO; green: laminin SMIFH2 vs. free-floating SMIFH2; yellow: laminin SMIFH2 vs. laminin DMSO; pink: free-floating SMIFH2 vs. free-floating DMSO. Note the absence of differentially expressed genes in SMIFH2 comparisons (yellow and pink). Dashed lines indicate log2FC = ±1 and *P*-adj = 0.05. (*B*) Volcano plot highlighting selected differentially expressed genes between laminin and free-floating conditions using the combined data (*Materials and Methods*). (*C*–*F*) Plots highlighting expression levels of specific gene categories: laminin-binding integrins (*C*), FH2 domain-containing formins (*D*), focal adhesion regulators (*E*), and Hippo pathway components together with their canonical transcriptional readouts (*F*). Colored points indicate genes with *P*-adj < 0.05. Orange points in *C*–*F* highlight genes of interest within each category (only those with *P*-adj < 0.05 are labeled). Dashed lines indicate log2FC = ±1.

To distinguish whether formin inhibition blocks elongation by disrupting cell fate or by removing mechanical force, we performed a transcriptomics analysis (bulk RNA-seq) comparing the effects of laminin substrate and SMIFH2 treatment (*Materials and Methods*). We identified differentially expressed genes when gastruloids were plated on laminin, regardless of SMIFH2 treatment (Fig. 5*A* and *SI Appendix*, Fig. S14). However, comparing DMSO- and SMIFH2-treated gastruloids revealed no differentially expressed genes, whether cultured under free-floating conditions or on laminin (Fig. 5*A* and *SI Appendix*, Fig. S14 and Table S1). Given the transcriptional equivalence between SMIFH2- and DMSO-treated samples we pooled them together to increase the statistical power of the analysis (n = 6 replicates per condition; see *Materials and Methods*), and performed differential expression analysis on the combined data (Fig. 5*B* and *SI Appendix*, Table S1). These results indicate that formin inhibition blocks axis elongation on laminin without detectable effects on bulk gene expression, consistent with a role based primarily on protrusive cellular behavior.

### Laminin Biases Cells Toward Migratory States While Preserving Axial Identity.

Next, we examined the gene expression changes associated with gastruloids growing on laminin. We observed changes in adhesion-related genes, including upregulation of the main laminin-binding integrins (Fig. 5*C*), several FH2 domain-containing formins including Fmn1, Fmn2, Fmnl2, and Fmnl3 (Fig. 5*D*), and focal adhesion (FA) regulators (Fig. 5*E*). Generally, we find that laminin increases the expression of factors related with the assembly of filopodia, stress-fibers and lamellipodia (*SI Appendix*, Fig. S15). Notably, while these expression changes are statistically significant, their magnitude is modest (generally with |log2FC| < 1). These changes in the bulk transcriptomics could come from modest widespread changes, or by more substantial changes in small cell populations. Regardless, free-floating gastruloids express formins and extend filopodia (*SI Appendix*, Fig. S12), yet achieve elongation through substrate-independent mechanisms that are insensitive to SMIFH2.

The transcriptional changes on laminin could be triggered by physical parameters associated with substrate adhesion, such as cell spreading. Consistent with this idea, we observe the upregulation of canonical YAP transcriptional targets (Ccn1, Ccn2, Myc; Fig. 5*F*), suggesting substrate-induced mechanotransduction consistent with previous reports linking cell spreading to YAP-dependent FA gene expression (38).

Beyond adhesion and cytoskeletal changes, laminin also influenced developmental signaling. Gene Ontology (GO) analysis of the differentially expressed genes (|log2FC| > 1 and *P*-adj < 0.05) shows changes in Wnt signaling (*SI Appendix*, Fig. S16): gastruloids on laminin showed upregulation of Wnt ligands and downregulation of Wnt antagonists, Nodal pathway components, and Shh targets (*SI Appendix*, Fig. S17 *A*–*D*). This transcriptional profile suggests laminin promotes a more posterior, mesenchymal identity consistent with the enhanced migratory behavior observed in these conditions, while driving the downregulation of more anterior endodermal fates (*SI Appendix*, Fig. S17 *E* and *F*). However, Hox gene expression remained largely unchanged (*SI Appendix*, Fig. S18), indicating that free-floating and laminin gastruloids acquire equivalent axial identities (posterior trunk) despite their distinct morphogenetic trajectories. Overall, the transcriptomic analysis shows that laminin biases cells toward migratory fates while the gastruloid preserves its axial identity. Critically, elongation on laminin selectively requires formin/myosin-dependent activity, a requirement that is dispensable in free-floating conditions, suggesting that formins play a role in elongation that only manifests in the presence of a substrate.

### Focal Adhesion Activity Is Required for Elongation on Laminin.

Having established that formin-dependent filopodia are essential for cell stream formation specifically on laminin, we next investigated the role of FAs in this process. FAs serve as mechanical linkages between the ECM and the actin cytoskeleton, transmitting traction forces required for cell migration on substrates. Given the upregulation of FA regulators we observed in our transcriptomics analysis (Fig. 5*E*), we hypothesized that FA-mediated traction forces are specifically required for axis elongation on laminin.

**Fig. 6.**
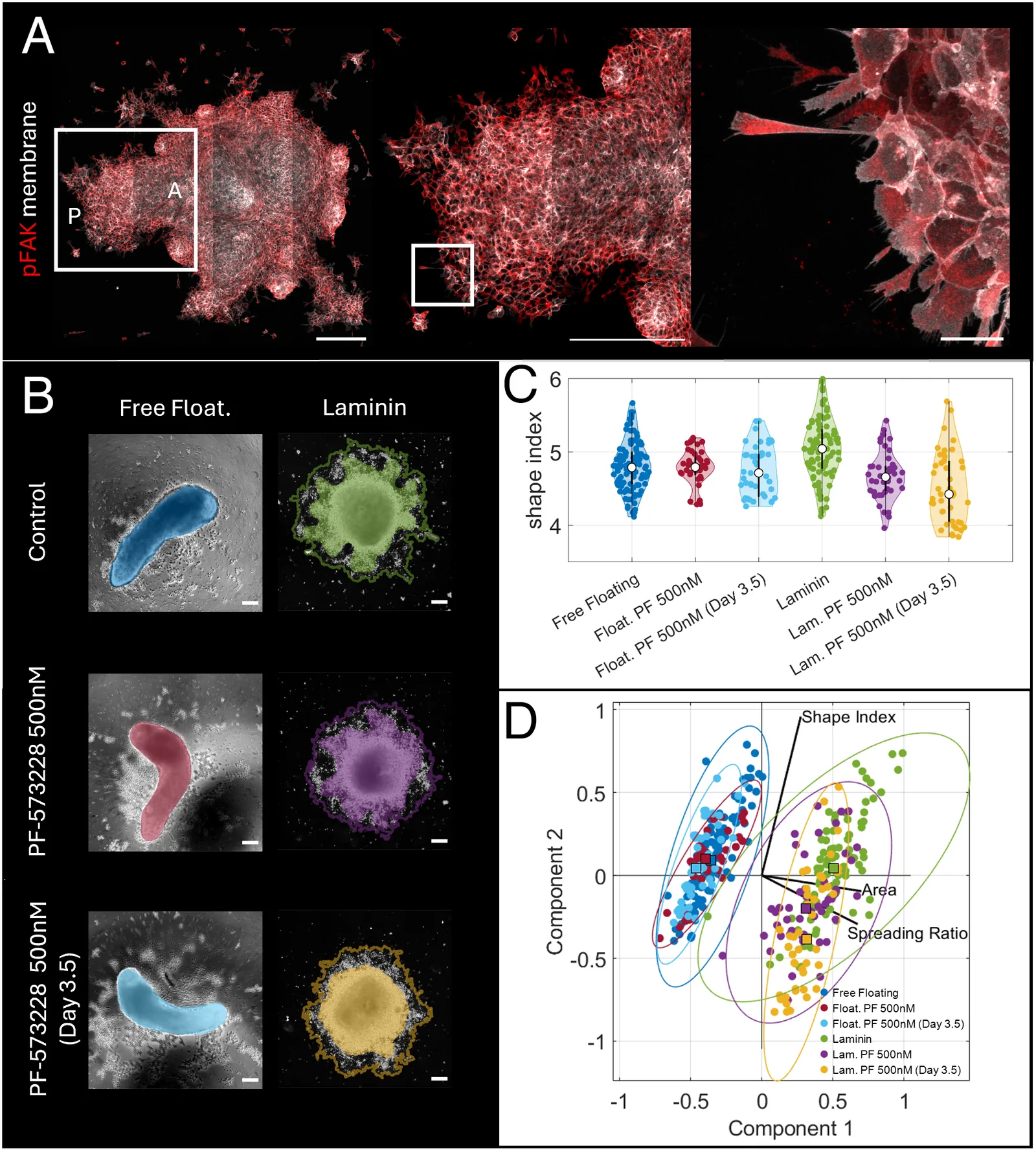
Focal adhesions are required for axis elongation only on laminin. (*A*) Immunostaining for phosphorylated FAK (pFAK, red) and membrane marker (white) in gastruloids cultured on laminin at day 5. pFAK is enriched at the gastruloid periphery, within cell streams, and in cellular protrusions. Boxes indicate magnified regions. A and P indicate the anterior pole (main body of the gastruloid) and posterior pole (tip characterized by protrusive activity and elongated morphology). (*B*) Representative images of free-floating and laminin gastruloids treated with DMSO (Control) or the FAK inhibitor PF-573228 (500 nM) added at day 4 (96 h) or day 3.5 (84h). Colored overlays indicate gastruloid outlines. (*C*) Quantification of shape index across conditions. FAK inhibition reduces morphological complexity specifically on laminin, with earlier treatment (day 3.5) showing stronger effects. (*D*) PCA. FAK inhibition shifts treated laminin gastruloids while free-floating gastruloids show no effect. See Dataset S1 for full statistical analysis. [Scale bars, 200 µm (*A*, *Left* and *Center*), 20 µm (*A*, *Right*), and 200 µm (*B*).]

We first examined the localization of phosphorylated Focal Adhesion Kinase (pFAK), a marker of active focal adhesions, in gastruloids cultured on laminin. pFAK was enriched at the periphery of the gastruloid, within the elongating cell streams, and in cellular protrusions (Fig. 6*A*), consistent with active traction force generation in these regions.

To functionally test the requirement for FAs, we treated gastruloids with PF-573228, a selective FAK inhibitor. FAK inhibition significantly blocked cell stream formation on laminin, with severity increasing with treatment duration, while free-floating gastruloids retained their normal elongated morphology (Fig. 6 *B*–*D*).

These results suggest that elongation on laminin requires focal adhesion activity, consistent with a requirement for substrate traction. Notably, FAK is highly expressed in both conditions (Fig. 5*E*, log2FC = 0.08), yet free-floating gastruloids do not need it for elongation. This suggests that morphogenetic plasticity could arise from the context-dependent engagement of preexisting cytoskeletal components.

## Discussion

Our findings reveal that gastruloids possess a temporal cell–substrate adhesion profile that mirrors the sequential deposition of ECM components in the embryo. Gastruloids show strong affinity for laminin from the time of aggregation, independent of Chiron treatment, likely reflecting the primordial role of laminin in mammalian gastrulation: It is the first ECM protein to appear in mammalian embryos (12, 24) and the only ECM component whose absence results in lethal phenotypes during gastrulation (39). In contrast, the delayed, Chiron-dependent adhesion to fibronectin and collagens may reflect the requirement for mesoderm-associated EMT and subsequent ECM-specific integrin expression (20).

Early attachment to laminin constrains gastruloid morphogenesis, preventing the formation of a single body axis. While free-floating gastruloids consolidate mesoderm precursors into a single pole through extensive cell rearrangements (13, 15, 40), gastruloids plated on laminin develop multiple elongating structures. The number of protrusions varies between individual aggregates, possibly because of a heterogenous induction process that does not always result in mesoderm sites at the periphery. This likely reflects the tight regulation exerted in vivo by other external factors like the geometry of the tissue or the extraembryonic tissues to give rise to a single body axis. We hypothesize that the multipolar morphology observed on laminin possibly arises because strong cell-substrate adhesion restricts cell mobility during days 2 to 4, trapping T/Bra-positive cells at their sites of induction around the gastruloid periphery. Unable to coalesce through collective rearrangements, each cluster of mesoderm precursors instead initiates local axis elongation, with each stream establishing proper anteroposterior patterning (Cyp26a1 at tips, Aldh1A2 in the body). Hox gene expression remains similar between conditions, indicating that both laminin and free-floating gastruloids acquire equivalent positional identity.

Our transcriptomics reveals upregulation of laminin-binding integrins, focal adhesion regulators, and canonical YAP transcriptional targets, consistent with substrate-induced mechanosensing (38). In teleost embryos, a similar Yap-dependent program of focal adhesion and actin regulators sustains the directed mesenchymal migration that assembles the body-axis (41). We also observe enhanced Wnt signaling on laminin. This coactivation of YAP and Wnt pathways is consistent with evidence that ECM mechanical properties can modulate Wnt/*β*-catenin signaling through integrin-dependent mechanisms, and that YAP/TAZ can transcriptionally regulate Wnt pathway components (42). The resulting enhancement of Wnt signaling correlates with downregulation of anterior primitive streak derivatives, including definitive endoderm markers, and upregulation of mesenchymal markers, suggesting that mechanotransduction on laminin biases cells toward posterior migratory fates while maintaining trunk identity as marked by Hox expression. Finally, blocking tissue elongation on laminin through formin inhibition does not result in transcriptional changes, suggesting that the transcriptional programs driving cell fate specification are robust to disruption of morphogenesis (43).

By day 4, mesoderm cells have acquired the molecular machinery necessary for substrate-based migration, enabling the transformation of mesoderm clusters into actively elongating streams. This morphogenetic process shows specific cytoskeletal requirements: Formin-dependent actin structures are essential for stream formation, while Arp2/3 activity paradoxically restricts elongation efficiency. A similar cytoskeletal signature has been described in glioma cells: When cultured under confined conditions on laminin tracks, individual cells switch to a collective linear migration mode in which formin inhibition decreases linear migration while Arp2/3 inhibition increases it (44). In line with our results, this balance was interpreted as a trade-off between formins, required to build the bundled actin cables that drive directional process extension, and Arp2/3-dependent lamellipodia that produce exploratory protrusions antagonizing committed migration. Furthermore, genetic removal of Arp2/3 (45) or its pharmacological inhibition with CK666 (46) has been shown to induce a lamellipodia-to-filopodia transition. Consistent with this, we observe a modest but significant increase in filopodia length upon CK666 treatment (*SI Appendix*, Fig. S9*C*), which may further contribute to the enhanced elongation of gastruloids cultured on laminin. In contrast, free-floating gastruloids are unaffected by inhibition of either formins or Arp2/3, indicating they employ different force-generation mechanisms: likely cell–cell interactions based on differential adhesion and cortical contractility (14, 16, 25, 28).

The substrate-specific phenotype of formin or FAK inhibition, in combination with the absence of transcriptional effects following formin inhibition, suggests that these proteins have primarily a mechanical function that only manifests in substrate-based morphogenesis. Formins are necessary for the formation of filopodia and stress fibers, both actin structures with important roles in cell adhesion and migration (*SI Appendix*, Fig. S15). However, we note that SMIFH2 also inhibits some myosins (37), including myosin 10, which localizes to filopodia tips, so the precise contribution of filopodia vs. other formin-dependent structures remains to be determined. Nevertheless, ROCK inhibition, which blocks myosin 2-dependent stress fiber contractility (47), impairs elongation in both free-floating and laminin conditions (*SI Appendix*, Fig. S6). These results, in combination with the ones above, indicate that the laminin-specific requirement involves formin-dependent actin nucleation rather than myosin 2-driven contractility. In addition, ROCK inhibition also increases the halo of individually migrating cells on laminin (*SI Appendix*, Fig. S6*B*), suggesting that ROCK-mediated contractility is required preferentially for the long-range coordination of collective stream migration rather than for individual cell motility (48, 49).

Nevertheless, focal adhesions can form at filopodia tips during collective cell migration (50), providing mechanical anchorage for force transmission. Furthermore, FAK-null mice can implant and initiate gastrulation; however, body-axis elongation was dramatically retarded by E8.5 (51). FAK deficiency did not impair the differentiation potential of mesoderm cells or their proliferation, but produced a drastic reduction in their migratory speed and directionality (52). Notably, FAK-deficient cells exhibited an increased number of focal adhesions rather than fewer, indicating that FAK promotes focal adhesion turnover rather than assembly (52). This is consistent with the failure of cell stream formation we observe upon FAK inhibition on laminin, where cells remain attached to the substrate but fail to elongate. Together, these results suggest that substrate-based mesoderm migration contributes to body-axis elongation in the embryo.

Notably, T/Bra-positive cells within free-floating gastruloids generate active protrusions dependent on noncanonical Wnt signaling (14, 53). Blocking noncanonical Wnt prevents coalescence of T/Bra clusters (14), suggesting this pathway drives the mechanical changes underlying mesoderm progenitor sorting. Together with our findings, this might suggest that noncanonical Wnt induces an excitable cytoskeleton that generates protrusions and alters mechanical properties, driving tissue elongation through cell–cell interactions in free-floating conditions or cell–substrate interactions when adherent to ECM.

Although the plastic properties of vertebrate gastrulation can be studied in the embryo (1, 54, 55), embryonic development is a tightly regulated, robust process that may mask the latent morphogenetic capacities of embryonic tissues (4). For these reasons, in vitro systems, where embryonic constraints are relaxed, may be better suited to reveal this hidden potential. For instance, cerebral organoids generate ventricle-like lumens through cavitation (56), whereas in vivo these structures derive from the neural tube lumen, which forms through folding morphogenesis. Yet when appropriate geometrical and adhesive constraints are applied, folding-based neurulation can be recapitulated (3). Analogously, mouse embryonic stem cell (mESC) aggregates normally form lumens through hollowing in an E-cad-dependent process, but in the absence of E-cad they generate lumen-like cavities via a closure mechanism instead (57).

Together with these examples, our findings demonstrate that cells retain the capacity for multiple morphogenetic programs to achieve structurally similar outcomes, perhaps through a process reminiscent of phenotypic accommodation (58). For instance, sea urchin blastulation normally occurs through blastocoel inflation; however, separated half-embryos at the two-blastomere stage form a functional blastula through an alternative route, folding into a cup before rounding into a hollow sphere (59). This flexibility may have evolutionary significance: It challenges deterministic views of morphogenesis and suggests that evolutionary innovations may arise not from new gene-regulatory programs, but from environmentally induced shifts in how existing cytoskeletal machinery is used, revealing latent mechanical capabilities without requiring genetic novelty.

**Fig. 7.**
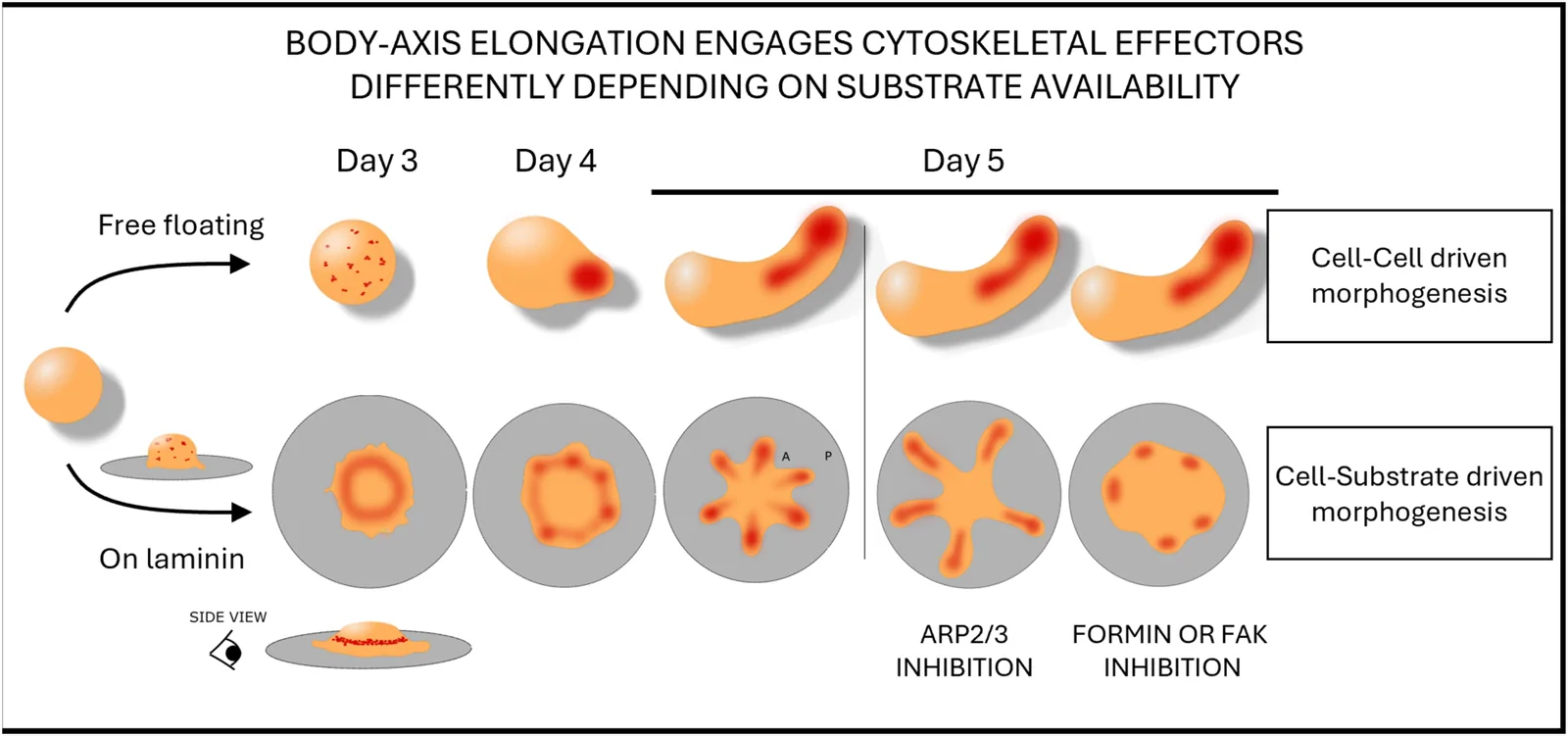
Adherent substrates reveal different morphogenetic routes to generate a posterior body axis in gastruloids. Summary of the findings: when grown on an adhesive substrate, gastruloids elongate using cell–substrate interactions regulated by Arp2/3, formins and focal adhesions. In contrast, free-floating gastruloids elongate independently of substrate contact, through mechanisms that do not require these cytoskeletal effectors.

However, these previous studies identified alternative morphogenetic outcomes without systematically comparing the cellular requirements of each mode. Here, we show that gastruloid elongation can proceed through alternative morphogenetic modes, and by applying the same perturbations across both conditions, we demonstrate that each mode has distinct cytoskeletal dependencies (Fig. 7). Understanding that morphogenetic outcomes can be plastically redirected by tuning mechanical context suggests new strategies for tissue engineering through the control of physical properties and cytoskeletal activity.

In conclusion, our work demonstrates that gastruloids can achieve body-axis elongation through different morphogenetic strategies depending on whether or not cells engage substrates to generate force. This shows that similar developmental outcomes can emerge from distinct force-generation strategies, providing potential raw material for evolutionary diversification. On the practical side, these findings suggest that desired morphologies might be achieved by triggering latent morphogenetic programs.

## Materials and Methods

Antibodies used are listed in *SI Appendix*.

### Cell Culture.

mESC were cultured at 37 °C, 5% CO_2_ in 2i LIF [N2B27 (NeuroBasal medium, DMEM F-12 no glutamine, N2—homemade (60)—B27, 2-Mercaptoethanol), L-glutamine, 3 µM CHIR99021, 1 µM PD0325901, and 0.1 µg mL^−1^ LIF]. All lines were cultured in filter-capped tissue culture flasks coated with 0.1% gelatin. Cell culture media was changed every day, and cells were passaged every 2 d. Cells were routinely tested for mycoplasma.

### Cell Lines.

Three mESC lines were used in this study. The wild-type line is ES-E14TG2a (E14; 129/Ola background; Merck). The SBN line is a triple reporter derived from CGR8 mESCs (129/Ola), carrying the Sox1-P2A-eGFP and Bra-P2A-mCherry reporters (61), which we further modified with a constitutive H2BmiRFP670 nuclear marker. The GPI-GFP line is derived from R1 mESCs (129X1/SvJ ×129S1/SvImJ) and carries the CAG::GFP-GPI plasma-membrane reporter transgene (62). We note that, when compared with the SBN, the GPI-GFP line cultured on laminin from the same number of starting cells (300) produces smaller gastruloids on day 5 (SBN: ~0.8 mm^2^, GPI-GFP: ~0.5 mm^2^) and reduced number of cell streams (SBN: 3 to 6 protrusions, GPI-GFP: 1 to 3 protrusions typically).

### Gastruloid Culture.

Gastruloids were generated following a previously published protocol (63). A total of 200 to 300 mESCs were aggregated in 40 µL of N2B27 medium supplemented with Penicillin-Streptomycin (1%) and L-glutamine (referred to as supplemented N2B27) using a cell-repellent, U-bottom 96-well plate (650970, Greiner). Cells were allowed to aggregate for 48 h, after which 150 µL of supplemented N2B27 containing 3 µM CHIR99021 (Chiron pulse) was added to each well. Gastruloids were incubated in this medium for 24 ± 2 h, after which 150 µL of medium was replaced daily with fresh supplemented N2B27. Gastruloids were maintained in culture for a total of 5 d. For the Activin A experiment, the Chiron pulse from the original protocol was replaced by an Activin A (100 ng/mL) pulse of 24 h, from 48 to 72 h.

### 2D Gastruloid Protocol.

Gastruloids were generated as described above. On day 2, 35 mm glass-bottom dishes were coated with 14 µg/mL Laminin 111 (L2020, Sigma) for 1 h at 37 °C. Laminin solution is prepared by diluting the stock solution in PBS without calcium and magnesium (PBS^−/−^) to a final concentration of 14 µg/mL. Gastruloids were collected using a cut 200 µL pipette tip and allowed to settle at the bottom of an Eppendorf tube for a few seconds. The medium was then carefully removed, and gastruloids were resuspended in supplemented N2B27 medium containing 3 µM CHIR99021. They were subsequently transferred, using a cut tip, into the Laminin-coated dish containing fresh medium. To prevent fusion, individual gastruloids were gently separated from one another using a cut tip. The dish was then placed in the incubator, which remained closed for at least 1 h to allow for proper attachment without disturbance. After 24 h, gastruloids typically appeared flatter. From this point, medium was changed daily as described above until day 5. In the same manner, fibronectin coating is realized by diluting Fibronectin stock solution in PBS^−/−^ to a final concentration of 12.5 µg/mL. The solution is transferred to a glass-bottom dish (400 µL for a 20 mm dish) and incubated for 1 h at 37 °C. Before seeding, the solution is removed from the dish and washed once with PBS^−/−^. For collagens coatings, solutions are kept on ice until use. Collagen I is prepared by diluting the stock solution in 0.02 N acetic acid to a final concentration of 12.5 µg/mL overnight at 4 °C. The solution is then added to the dish and left to incubate at RT for 1 h. Dishes are rinsed twice with PBS to remove acid prior to gastruloid plating. Collagen IV is prepared by diluting the stock solution in cold deionized water to a working concentration of 12.5 µg/mL. The subsequent steps are identical to those described for Collagen I. For the ratio experiment, “100%” conditions of Collagen IV and Laminin are prepared by diluting proteins to a final concentration of 14 µg/mL in PBS. The mixtures (Laminin 75/Collagen 25, Laminin 50/Collagen 50, etc.) are prepared to reach the same total concentration of 14 µg/mL (100%). Coatings were left 1 h at 37 °C before seeding of the gastruloids.

### Mosaic Generation.

Mosaics were generated following the 3D gastruloid protocol described above but using a cell suspension composed of 95% E14 and 5% GPI-GFP cells.

### Chemical Perturbations.

Inhibitor treatments were carried out from 84 or 96 h. CK666 (SML0006-5MG, Sigma) is resuspended in DMSO at a 10 mM concentration upon reception and stored at 4 °C. SMIFH2 (HY-16931, MedChemExpress) is resuspended in DMSO at a 10 mM concentration upon reception and stored at −80 °C. GSK269962A (S7687-SEL, Selleckchem) is resuspended in DMSO at a 10 mM concentration upon reception and stored at −80 °C. PF-573228 (HY-10461, MedChemExpress) is resuspended in DMSO at a 10 mM concentration upon reception and stored at −80 °C. Inhibitor concentrations are as follows: CK666 (10 and 50 µM), SMIFH2 (10 and 25 µM), GSK269962A (0.1 µM), and PF-573228 (500 nM).

### Immunofluorescence Staining.

Gastruloids for staining are transferred to an Eppendorf using a cut tip, they are then washed once with PBS^−/−^ before being fixed with 4% PFA overnight at 4 °C (or 3 h at room temperature). The 2D gastruloid staining protocol is identical to the 3D protocol, except that all the steps are performed directly in the dish. On the following day, gastruloids are washed twice with PBS^−/−^, prewashed with PBSFT (PBS^−/−^ with 10% FBS (F9665-500 ML, Sigma) and 0.2% Triton X-100), washed three times with PBSFT at room temperature (10 min), and incubated in PBSFT for 1 h at 4 °C on an orbital shaker for blocking. Primary antibody solutions are prepared in PBSFT and used to replace the blocking solution once blocking is complete. Gastruloids are incubated overnight at 4 °C on an orbital shaker. For 2D gastruloids, to avoid wasting antibodies, the full dish is not filled with solution. Instead, a dome of antibody solution covering the glass part of the dish is sufficient (around 400 µL for a 20 mm diameter glass-bottom dish). Next day, the primary antibody solution is washed as follows: 2 × 5 min in PBSFT (RT), 3 × 10 min PBSFT (4 °C, orbital shaker), 4 × 30 min (4 °C, orbital shaker). Secondary solutions are prepared in PBSFT with nuclear (Sytox/DAPI) or/and Phalloidin. PBSFT wash is replaced with secondary solutions and the tubes are covered in foil and incubated overnight at 4 °C on an orbital shaker. Next day, secondary solutions are washed as described for the primary solutions. 3D gastruloids are placed in clearing solution—Scale S4, (64)—for 24 h before mounting. 2D gastruloids are directly imaged or stored in PBS and sodium azide (0.05%) until imaging. A list of the antibodies used in this study can be found in *SI Appendix*.

### Hybridization Chain Reaction.

Fixed gastruloids are dehydrated in methanol as follows: 5 min per wash at room temperature: 25% MetOH/75% PBST (PBS^−/−^, 0.2% Triton X-100), 50% MetOH/50% PBST, 75% MetOH/25% PBST, 100% MetOH twice. Gastruloids are then incubated overnight at −20 °C. Next day, probe hybridization buffer is prewarmed in the incubator and amplification buffer is brought at room temperature. Gastruloids are rehydrated with a series of 5-min washes, 75% MetOH/25% PBST, 50/50, etc. Once the last wash is removed, gastruloids are prehybridized using warm probe hybridization buffer for 30 min at 37 °C. Probe solution is prepared by adding 2 pmol of each probe set into hybridization buffer at 37 °C, the solution is transferred to the gastruloids and incubated overnight at 37 °C. Next day, prewarm probe wash buffer in the incubator and wash the gastruloids 4 × 15 min at 37 °C. Wash 2 × 5 min with 5 × SSCT at room temperature. Amplification buffer is added to the gastruloids to preamplify (5 min, RT). Prepare hairpin solutions following supplier’s recommendation and add the solution to the gastruloids, incubate overnight in the dark at room temperature. The next day, remove excess hairpins by washing 3 × 15 min with SSCT at room temperature. Samples can be stored at 4 °C until imaging/DAPI staining overnight in PBST. All HCR probes were bought from Sigma Aldrich.

### Confocal Microscopy.

Individual gastruloids were imaged using a Zeiss LSM700 confocal microscope, typically with a 20× objective, 0.5× zoom, and 2 µm axial slicing. For live-imaging experiments, we used a humidified incubation chamber (5% CO_2_, 37 °C) with a time step of 5 min for 12 h and an interval between slices of 2 µm.

### High-Throughput Microscopy.

Individual gastruloids were imaged using a Nikon Eclipse Ti-E inverted bright-field microscope equipped with a motorized stage, an Orca Flash 4.0 camera, a Lumencor SPECTRA X Light Engine with internal 391/22 nm, 475/28 nm, 555/28 nm, and 635/22 nm excitation filters and a humidified incubation chamber (5% CO_2_, 37 °C). To locate each sample, we performed tile scans of individual positions on a 96-well plate (2 × 2) or 35 mm glass-bottom dish (7 × 7) at 5× magnification. Gastruloid positions were then selected using our custom MATLAB-based tool, which enables loading of multipoint microscopy data and identification of gastruloids either manually through user selection or via optional AI-assisted detection powered by Cellpose segmentation, all through an intuitive graphical interface. Once individual gastruloid positions had been recorded, we acquired single snapshots or time-lapse images with a 20× objective, capturing phase contrast and fluorescence images with appropriate illumination channels as needed.

### Light-Sheet Microscopy.

Individual gastruloids for mosaic experiments (see above) were imaged on a Viventis LS2 light-sheet microscope (software v2.0.0.5) with dual-sided illumination (2 µm light sheet) and two opposing Nikon CFI75 16×/0.8 NA water-immersion detection objectives, whose views were fused during acquisition. Two channels were acquired sequentially at 100 ms exposure: 488 nm (50% power, LP 488 emission filter), and 638 nm (15%, 488/561/638 triple-band-pass). Three-dimensional renderings were generated in Imaris 9.2.1.

### High-Throughput Image Analysis.

We developed a MATLAB-based computational pipeline that integrates multiple tools for the segmentation and analysis of gastruloids. We used Cellpose models (65) trained from scratch to segment the main body of the gastruloid independently of the variable morphologies obtained in free-floating conditions or on ECM-coated dishes. To identify the area covered by migrating cells, we used the cell-probability map from a pretrained Cellpose 2 CP model and defined cell-covered area as all pixels with probability > 0.35. The spreading ratio is computed as the area covered by migrating cells outside the main body, divided by the area of the main body. For quantitative parameter extraction (area, elongation, circularity, fluorescence intensity, etc.), as well as the LOCO-EFA descriptors (33), we fed the Cellpose segmentations and original images into MOrgAna (66). Finally, diagnostic visualization, data representation, and statistical analysis were performed using MATLAB. Code for this pipeline can be accessed in https://github.com/gserranonajera/high-throughput-gastruloid-analysis.

### Particle Image Velocimetry.

Velocity fields were computed from confocal time-lapse movies (time frame = 5 min) using PIVLab v2.56 for MATLAB (67) with two passes of 64 × 64 and 32 × 32 pixel interrogation windows with 50% overlap with default pre- and postprocessing parameters.

### Lamellipodia and Filopodia Quantification.

Lamellipodia were manually delineated using the polygon tool in FIJI (68). Individual polygon coordinates were exported using a custom ImageJ macro, and the area of each polygon (lamellipodia) was computed and visualized using MATLAB. Filopodia were measured through a similar procedure, using the free-line tool in FIJI to manually trace them. The tissue depth of individual filopodia was computed as the distance from the filopodium center to the nearest tissue boundary, determined by Otsu-based segmentation of the nuclear signal in MATLAB.

### Cell Division Angle Quantification.

Division angles between daughter cell pairs were quantified at the moment of cytokinesis using confocal time-lapse movies acquired between days 4 and 5 of gastruloid development on laminin-coated substrates. The quantification procedure involved the following steps:

1. Daughter cell pair coordinates were manually identified using the multipoint tool in FIJI, and the resulting coordinates were exported to MATLAB for analysis.
2. The main elongation axis of each cell stream was defined by drawing a line along the stream’s length on the final frame of the time-lapse movie using MATLAB’s drawline function.
3. Cell divisions were filtered to include only those occurring within the “neck” region of the cell stream, which was defined by manual delineation using MATLAB’s drawpolygon function.
4. The angle between each cell division axis and the main elongation axis of the corresponding cell stream was calculated for all samples and experimental conditions.

### Statistical Analysis.

All results were compiled into matrices showing adjusted *P*-values for all pairwise comparisons and exported as Excel files. See Dataset S1 to find the statistical analysis associated with each figure.

#### Analysis of data from the high-throughput pipeline.

Statistical analyses were performed using MATLAB using a nonparametric approach. The Wilcoxon rank-sum test (Mann–Whitney U test) was conducted between all possible group combinations. This nonparametric test ranks all observations from both groups being compared and tests whether the distributions differ significantly, making it suitable for data that may not meet the assumptions of parametric tests. To account for multiple comparisons in the pairwise testing phase, *P*-values were adjusted using the Bonferroni correction method, where each *P*-value was multiplied by the total number of pairwise comparisons and capped at 1.

#### Analysis of lamellipodia and filopodia.

MATLAB’s implementation of the Wilcoxon rank-sum test was conducted between all possible group combinations. *P*-values were adjusted using the Bonferroni correction method.

#### Analysis of cell division angle data.

Cell division angle analysis was performed using circular statistics appropriate for axial data using the Circular Statistics Toolbox (69) for MATLAB. Division angles were measured as the angle between the division axis and a reference orientation, where angles *θ* and *θ* + *π* represent the same division axis. To apply circular statistical methods to axial data, all angles were converted to circular representation by doubling (*θ →* 2*θ*), transforming the axial period of *π* to a circular period of 2*π*. This transformation preserves the axial symmetry while enabling the use of standard circular statistical functions. To test the alignment of the distribution with a preferred direction, the V-test (Rayleigh test for a specified direction) was used to test for significant concentration around specific orientations. This nonparametric test evaluates the null hypothesis that data are uniformly distributed against the alternative hypothesis of concentration around a specified direction. We tested both groups for alignment at 0 (horizontal divisions) and *π*/2 (vertical divisions) orientations with respect to the elongation axis.

### Computational Surface Extraction.

Active myosin resides in the most apical compartment of the cells. For this reason, maximum projections may not accurately represent the apical myosin, including the myosin cables. To tackle this problem we computationally acquire a 2D surface of the most apical side of the sample. To extract the apical surface of the gastruloid from the confocal image volumes, we used a custom MATLAB script based on the square gradient focusing algorithm (55). The steps are as follows:

1. Tile the confocal volume into columns.
2. Apply the square gradient focusing algorithm to each column to find the surface position.
3. Generate a height map by finding the depth of the fastest change in the image sharpness (peak of the second derivative) for each column.
4. Apply a smoothing filter to the height map.
5. Use the smoothed height map to section the image volume and produce a 2D image of the tissue surface.

### Transcriptomics Analysis.

#### Experimental design and sample collection.

RNA-sequencing was performed on gastruloids cultured under four conditions: free-floating with DMSO (FF-DMSO), free-floating with SMIFH2 25 µM (FF-SMIFH2), laminin with DMSO (LM-DMSO), and laminin with SMIFH2 25 µM (LM-SMIFH2). For each condition, three biological replicates were collected at day 5 (120 h), with each replicate consisting of pooled gastruloids (n = 24 per replicate for free-floating, n = 20 per replicate for laminin). SMIFH2 or DMSO vehicle was added at 84 h. Gastruloids were collected, washed in PBS, and lysed in TRIzol. Total RNA was extracted using phenol-chloroform phase separation followed by isopropanol precipitation.

#### Library preparation and sequencing.

RNA-sequencing libraries were prepared and sequenced at the Cambridge Stem Cell Institute sequencing facilities. All 12 samples were processed in a single batch. Starting from 300 ng of total RNA per sample, poly(A) mRNA was isolated using the NEB Poly-A Magnetic Isolation Module, which employs oligo d(T) beads to capture polyadenylated transcripts. External RNA controls (ERCC spike-ins) were included prior to library preparation. Strand-specific libraries were generated using the NEBNext Ultra II Directional RNA Library Prep Kit. Libraries were quantified, combined in an equimolar pool, and sequenced on an Illumina NovaSeq X platform using a paired-end 150 bp (PE150) configuration. Sequencing was performed to an average depth of approximately 30 to 50 million paired-end reads per sample. Base calling and demultiplexing were carried out by the sequencing facility prior to downstream bioinformatic analysis.

#### Bioinformatic processing.

Raw sequencing data were converted to FASTQ format using Illumina bcl2fastq software. Sequencing quality was assessed using FastQC, and quality summaries were compiled using MultiQC (70). Adapter trimming and quality filtering were performed using Trim Galore (v0.6.1) in paired-end mode, retaining reads with a minimum length of 25 bp (71). Posttrimming read quality was confirmed using FastQC. Trimmed reads were aligned to the mouse reference genome (mm10/GRCm38) using BWA-MEM (72), with the -M flag enabled. SAM files were converted to BAM format and processed using SAMtools v1.13 (73). Mate information was added using samtools fixmate, followed by coordinate sorting. PCR duplicates were identified and removed using samtools markdup -r, generating duplicate-filtered BAM files. Reads mapping to the mitochondrial chromosome (chrM) were excluded prior to downstream analysis. BAM files were indexed, and alignment quality was assessed using samtools flagstat at multiple processing stages (73). Gene-level quantification was performed using featureCounts, assigning uniquely mapped reads to annotated exons using the Ensembl Mus musculus GRCm38 gene annotation (74).

#### Differential expression analysis.

Differential gene expression analysis was carried out in R using DESeq2 (75). Raw counts were normalized using the median-of-ratios method, and lowly expressed genes were filtered prior to model fitting. To assess the transcriptional effects of both culture substrate and SMIFH2 treatment, four pairwise comparisons were initially performed using three biological replicates per condition: 1) LM-DMSO vs. FF-DMSO, 2) LM-SMIFH2 vs. FF-SMIFH2, 3) FF-SMIFH2 vs. FF-DMSO, and 4) LM-SMIFH2 vs. LM-DMSO. *P*-values were adjusted for multiple testing using the Benjamini–Hochberg method, and genes with an adjusted *P*-value below 0.05 were considered significantly differentially expressed. Log2 fold-change shrinkage was applied using DESeq2 shrinkage estimators for visualization and ranking.

Comparison of DMSO- and SMIFH2-treated samples revealed no differentially expressed genes in either free-floating or laminin conditions (Fig. 5*A* and *SI Appendix*, Fig. S14), confirming that SMIFH2 treatment does not alter the transcriptional state of gastruloids (*SI Appendix*, Table S1). Given this transcriptional equivalence, and that all samples were processed in the same sequencing batch, DMSO- and SMIFH2-treated samples were pooled within each culture condition to increase statistical power for detecting substrate-dependent gene expression changes. This resulted in six biological replicates per condition (free-floating vs. laminin). The pooled analysis yielded substantially improved statistical significance (Fig. 5*B* and *SI Appendix*, Table S1), confirming that DMSO- and SMIFH2-treated samples are not merely lacking detectable differences but are transcriptionally consistent within each culture condition.

### GO Analysis.

GO enrichment analysis was performed on genes with |log2FC| > 1 and adjusted *P*-adj < 0.05 using GeneCodis 4.0 (76).

### Gene Category Annotations.

To examine expression changes in specific functional categories, we curated gene lists from GO and published single-cell RNA-sequencing datasets.

#### GO-derived categories.

Genes associated with focal adhesion regulation, filopodium assembly, and stress fiber assembly were obtained by combining related GO Biological Process terms. For filopodium assembly, we merged genes annotated with the following terms: “filopodium assembly” (GO:0046847), “regulation of filopodium assembly” (GO:0051489), “positive regulation of filopodium assembly” (GO:0051491), and “negative regulation of filopodium assembly” (GO:0051490). Similar approaches were used for focal adhesion regulation and stress fiber assembly, combining parent terms with their positive and negative regulatory subterms. GO annotations were obtained from the org.Mm.eg.db R package.

#### Cell type markers.

Gut endoderm and mesenchyme marker genes were derived from the single-cell molecular atlas of mouse gastrulation (77). Differentially expressed genes defining gut endoderm and mesenchyme clusters at embryonic day 8.5 were extracted from the published dataset.

#### Other categories.

Laminin-binding integrins, FH2 domain-containing formins, Hippo transcriptional targets, and signaling pathway components (Wnt ligands, Wnt secreted antagonists, Nodal core signature, Shh targets) were manually curated from published literature.

### Manuscript Preparation.

During the preparation of this work, the authors used Claude Sonnet 4 in order to check grammar and readability. While using this tool/service, the authors reviewed and edited the content as needed and take full responsibility for the content of the publication.

## Supplementary Material

Appendix 01 (PDF)

Dataset S01 (XLSX)

Movie S1. Gastruloids on different ECM components from day 4 to 5. Red: T/Bra reporter (SBN line). Scale bar 500 μm.

Movie S2. Gastruloid on laminin from day 4 to 5. Cell membrane labelled with GFP (GPI-GFP line). Scale bar 500 μm.

Movie S3. Gastruloid on laminin from day 4 to 5 in the presence of CK666 50 μM. Cell membrane labelled with GFP (GPI-GFP line). Scale bar 500 μm.

Movie S4. Gastruloids on laminin from day 4 to 5 with CK666 at 25 and 50 μM. Red: T/Bra reporter (SBN line). Scale bar 500 μm.

Movie S5. Visualisation of cell divisions on an extending tip from gastruloid on laminin from day 4 to 5. All detected divisions are shown simultaneously at the end of the video. Cell membrane labelled with GFP (GPI-GFP line). Scale bar 100 μm.

Movie S6. Visualisation of cell divisions on an extending tip from gastruloid on laminin from day 4 to 5 in the presence of CK666 50 μM. All detected divisions are shown simultaneously at the end of the video. Cell membrane labelled with GFP (GPI-GFP line). Scale bar 100 μm.

Movie S7. Gastruloid on laminin from day 4 to 5 in the presence of SMIFH2 25 μM. Cell membrane labelled with GFP (GPI-GFP line). Scale bar 500 μm.

## Data Availability

Code data have been deposited in GitHub (78). All other data are included in the manuscript and/or supporting information.
