# Supplementary material for: Gastruloids reveal alternative morphogenetic routes for body-axis elongation with distinct cytoskeletal dependencies
Source: Proc Natl Acad Sci U S A. 2026 Nov 6;123(45):e2606813123. doi: 10.1073/pnas.2606813123 (PMC13752376; doi:10.1073/pnas.2606813123)
Supplement: Supplementary file 1 [file pnas.2606813123.sapp.pdf]

PNAS

**Supporting Information for**
Gastruloids reveal alternative morphogenetic routes for body axis elongation with distinct cytoskeletal dependencies

Corresponding authors: Guillermo Serrano Nájera and Benjamin J. Steventon

Email: guillermosn@mrclmb.ac.uk, bjs57@cam.ac.uk

**This PDF file includes:**

Figures S1 to S18
Table S1
Legends for Movies S1 to S7
Antibody list

**Other supporting materials for this manuscript include the following:**

Movies S1 to S7
Dataset S1 - Statistical analysis
Software:
https://github.com/gserranonajera/high-throughput-gastruloid-analysis

## Supplementary movies

- Movie 1 Gastruloids on different ECM components from day 4 to 5. Red: T/Bra reporter (SBN line). Scale bar 500 μm.
- Movie 2 Gastruloid on laminin from day 4 to 5. Cell membrane labelled with GFP (GPI-GFP line). Scale bar 500 μm.
- Movie 3 Gastruloid on laminin from day 4 to 5 in the presence of CK666 50 μM. Cell membrane labelled with GFP (GPI-GFP line). Scale bar 500 μm.
- Movie 4 Gastruloids on laminin from day 4 to 5 with CK666 at 25 and 50 μM. Red: T/Bra reporter (SBN line). Scale bar 500 μm.
- Movie 5 Visualisation of cell divisions on an extending tip from gastruloid on laminin from day 4 to 5. All detected divisions are shown simultaneously at the end of the video. Cell membrane labelled with GFP (GPI-GFP line). Scale bar 100 μm.
- Movie 6 Visualisation of cell divisions on an extending tip from gastruloid on laminin from day 4 to 5 in the presence of CK666 50 μM. All detected divisions are shown simultaneously at the end of the video. Cell membrane labelled with GFP (GPI-GFP line). Scale bar 100 μm.
- Movie 7 Gastruloid on laminin from day 4 to 5 in the presence of SMIFH2 25 μM. Cell membrane labelled with GFP (GPI-GFP line). Scale bar 500 μm.

## Dataset S1 - Statistical Analysis

All results were compiled into matrices showing adjusted p-values for all pairwise comparisons and exported as Excel files. See the Dataset S1 to find the statistical analysis associated with each figure.

## Supplementary Tables

**Supplementary Table 1.** Differentially expressed genes across conditions. Genes with adjusted p-adj $< 0.05$ and $|\log_2 FC| > 1$ were considered differentially expressed.

| Comparison | Replicates | Upregulated | Downregulated |
|---|---|---|---|
| *Effect of SMIFH2 treatment* | | | |
| Free-floating: SMIFH2 vs DMSO | 3 vs 3 | 0 | 0 |
| Laminin: SMIFH2 vs DMSO | 3 vs 3 | 0 | 0 |
| *Effect of substrate* | | | |
| Laminin vs Free-floating (DMSO only) | 3 vs 3 | 74 | 190 |
| Laminin vs Free-floating (SMIFH2 only) | 3 vs 3 | 55 | 181 |
| Laminin vs Free-floating (pooled) | 6 vs 6 | 116 | 266 |

**Supplementary Figures**

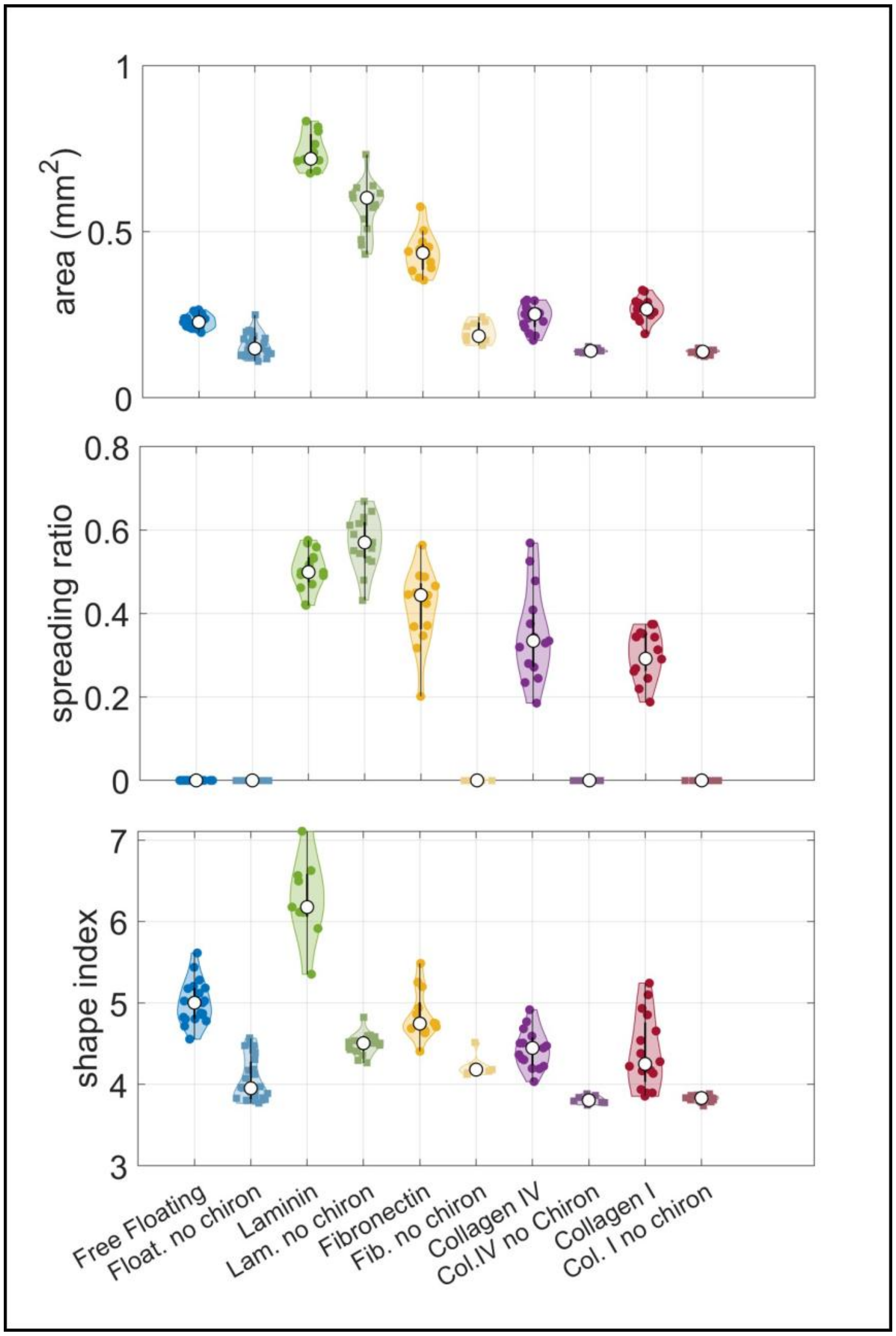


**Supplementary Fig. 1. Characterisation of gastruloids grown on different substrates.** Analysis of area, migratory abilities (spreading ratio) and elongation (shape index) on day 5 gastruloids grown on different coatings in the presence/absence of Chiron. See Dataset S1 for the full statistical analysis.

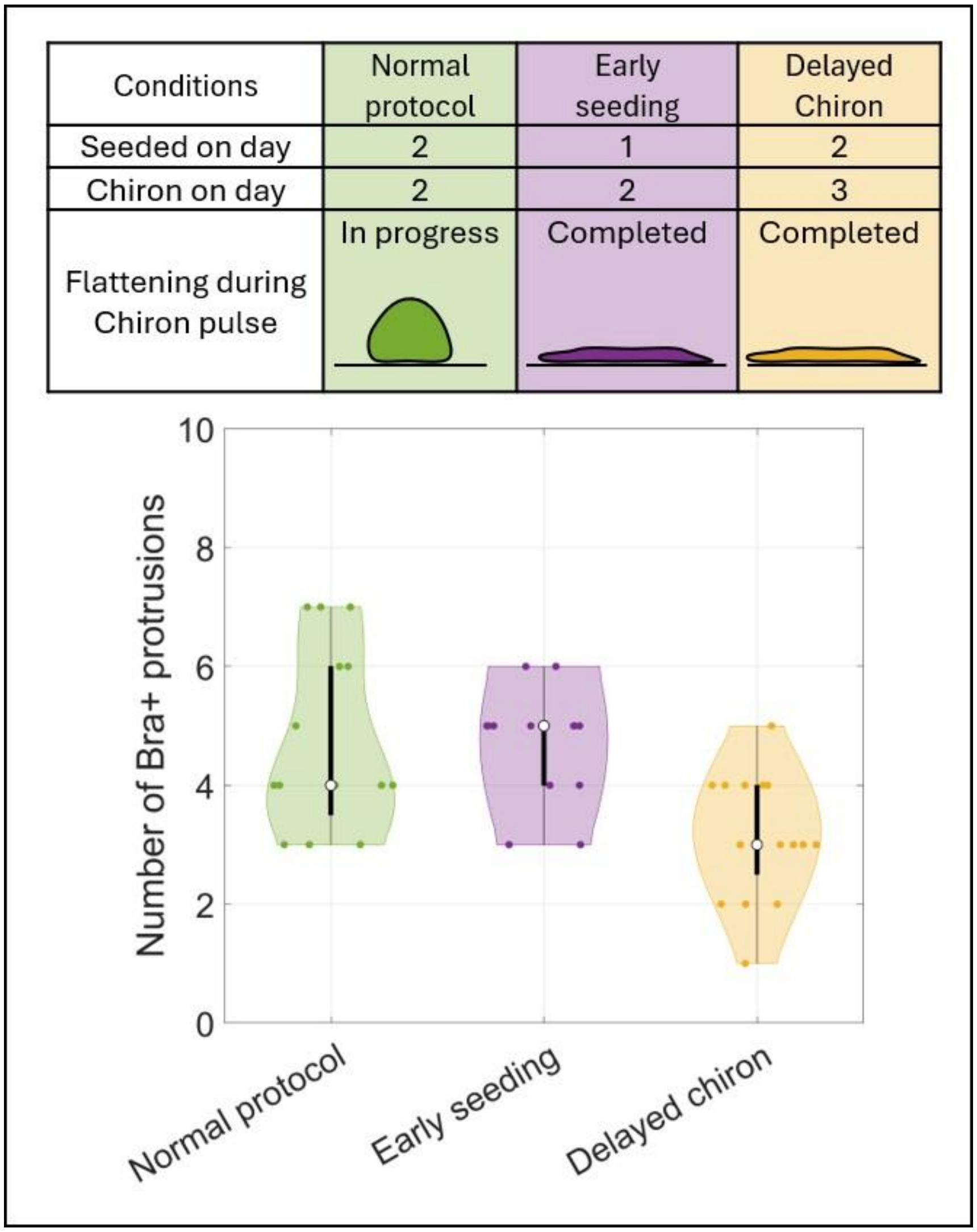


**Supplementary Fig. 2. Protrusion number is insensitive to tissue thickness at the time of the Chiron pulse but decreases when the pulse is delayed.** (Top) Schematic of the three conditions tested. In the normal protocol, gastruloids are seeded on laminin and receive the Chiron pulse on day 2, so flattening is still in progress during the pulse. In the early-seeding condition, gastruloids are seeded on day 1 and are fully flattened before Chiron is added on day 2. In the delayed-Chiron condition, gastruloids are seeded on day 2 but receive Chiron on day 3, after flattening is complete. Cartoons indicate the cross-sectional tissue profile (degree of flattening) at the time of the pulse. (Bottom) Number of T/Bra+ protrusions per gastruloid quantified on day 5 for each condition. See Dataset S1 for the full statistical analysis.

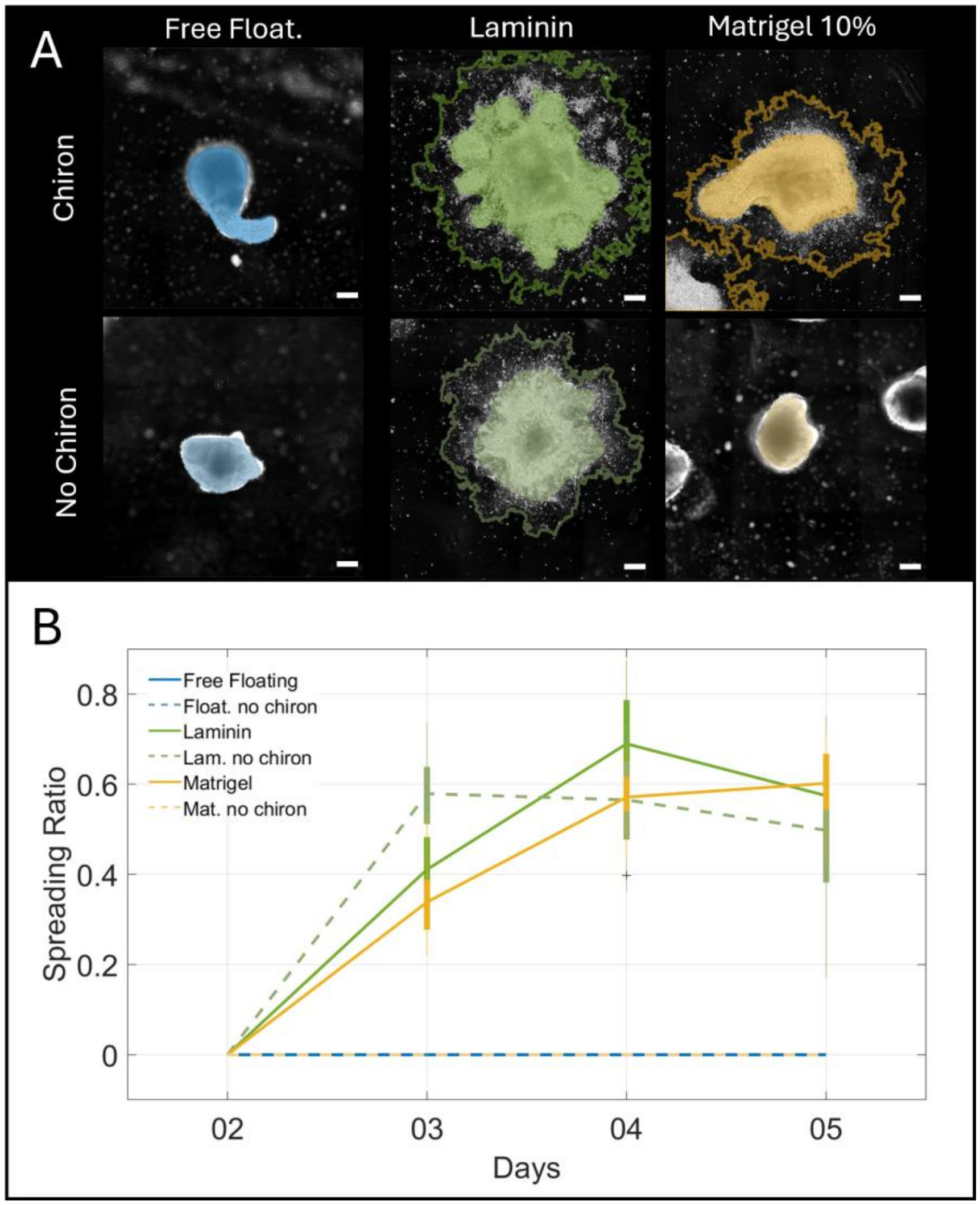


**Supplementary Fig. 3. Adhesion to Matrigel requires chiron.** A) Representative morphologies depending on the substrate and the presence of chiron. B) Analysis of the spreading capability of gastruloids on different substrates from day 2 to 5. Scale bars 200 $\mu m$.

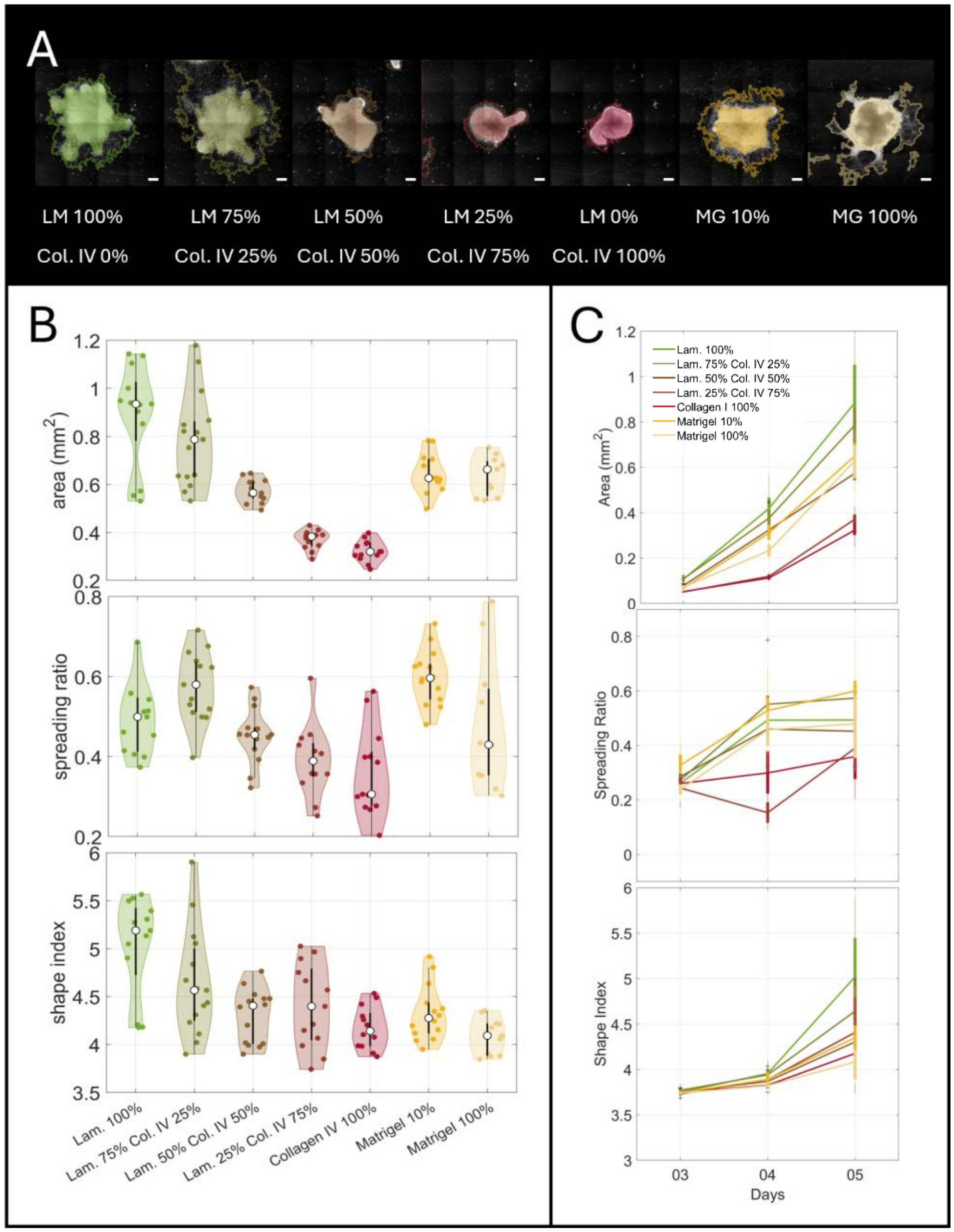


**Supplementary Fig. 4. Laminin-Collagen IV mixtures reproduce Matrigel's adhesion profile.** A) Representative morphologies depending on the substrate. B) Analysis of area, migratory abilities (spreading ratio) and elongation (shape index) on day 5 gastruloids grown on different coatings. C) Analysis of the spreading capability of gastruloids on different substrates from day 3 to 5. Scale bars 200 $\mu m$. See Dataset S1 for the full statistical analysis.

A

| | This study | Morgani et al., 2018 |
|---|---|---|
| Preconditioning | Aggregation in U-wells | EpiLC conversion (48 hr, Fibronectin, N2B27 + FGF2 + ACTIVIN A +1% KOSR) |
| Signalling protocol | Chiron pulse | FGF2 + ACTIVIN A + BMP4 + WNT3A |
| Substrate | Unrestricted laminin coating | Laminin-coated micropattern chips |

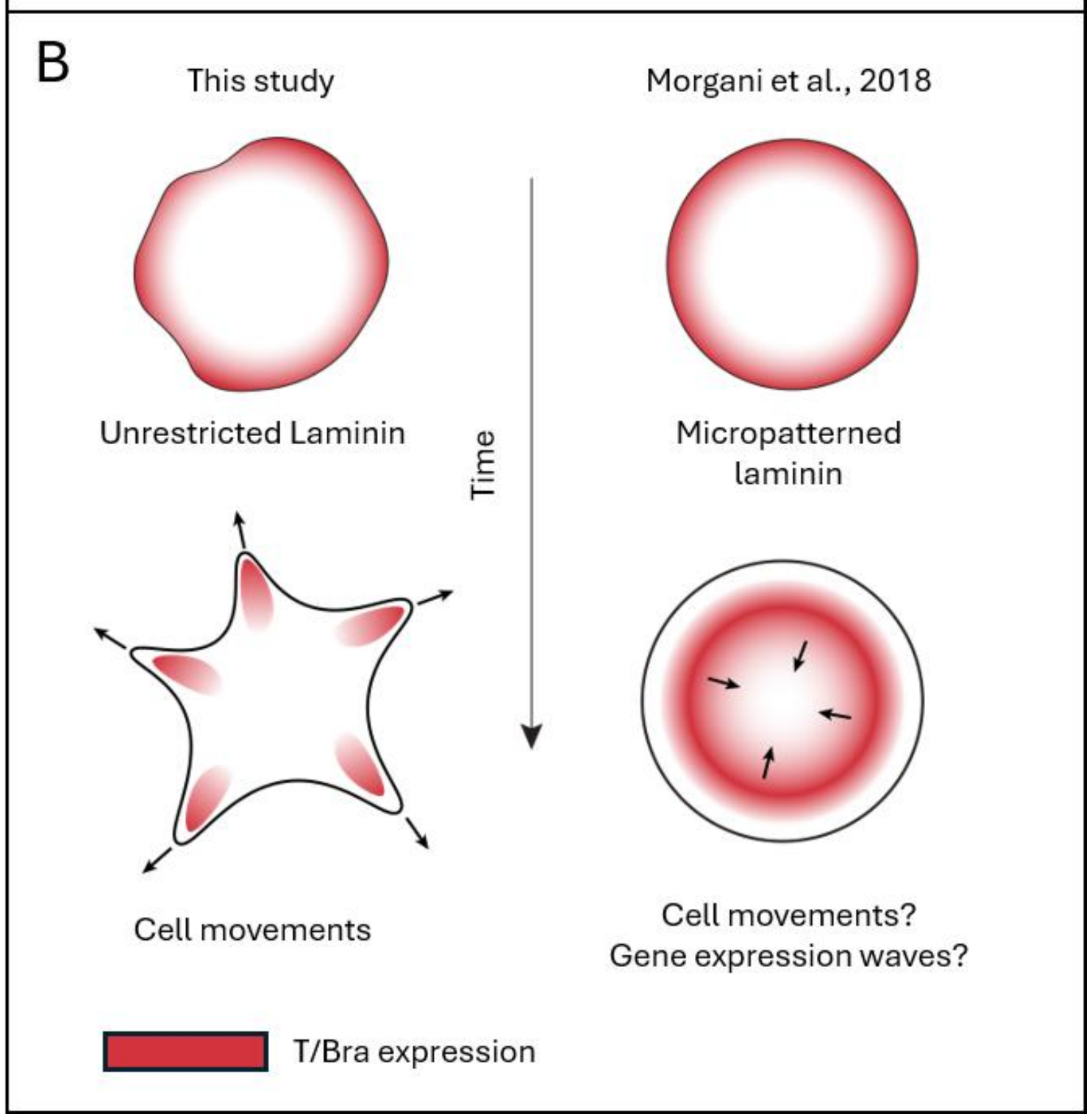


**Supplementary Fig. 5. Comparison of two in-vitro models of mouse posterior body-axis / mesoderm patterning.** (A) Summary of the key protocol differences between this study and the micropattern system of Morgani et al. (2018). In this study, mouse ESCs are aggregated, induced with a Chiron pulse, and cultured on an unrestricted laminin coating. In Morgani et al., ESCs are first converted to epiblast-like cells (EpiLCs), differentiated with exogenous FGF2, ACTIVIN A, BMP4 and WNT3A, and cultured on geometrically defined laminin-coated micropattern chips. (B) Schematic of T/Bra (red) dynamics over time. On unrestricted laminin (left), gastruloids initially show peripheral T/Bra and then break symmetry to form multiple elongating cell streams with T/Bra at their outward-moving tips, driven by collective cell migration. On micropatterned laminin (right), T/Bra is first induced at the colony edge and subsequently shifts toward the colony centre; in Morgani et al. it is left open whether this inward shift reflects cell movement, a propagating wave of gene expression, or both.

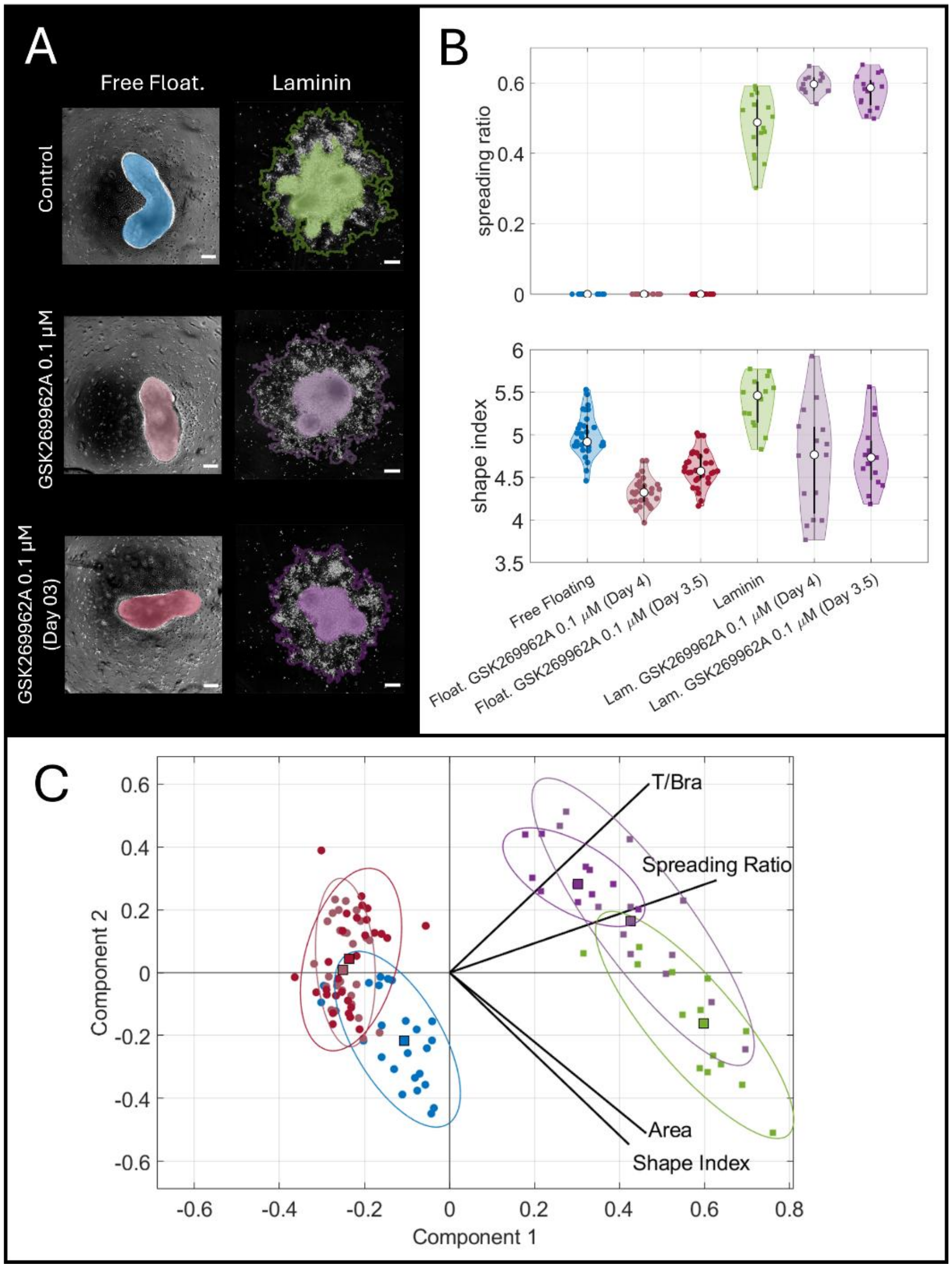


**Supplementary Fig. 6. Myosin contractility is necessary for elongation in free-floating and laminin gastruloids.** A) Gastruloid morphologies on different substrates with or without the ROCK inhibitor GSK269962A 0.1 $\mu M$ added on day 3 or 4. B) Top: Analysis of the spreading capabilities after ROCK inhibition. Cell migration is enhanced on laminin. Bottom: Quantification of the shape index after ROCK inhibition. Free-floating and laminin gastruloids show a reduced elongation. C) Principal Component Analysis integrating different properties on day 5. Note that treated free-floating gastruloids do not overlap with the controls. See Dataset S1 for the full statistical analysis.

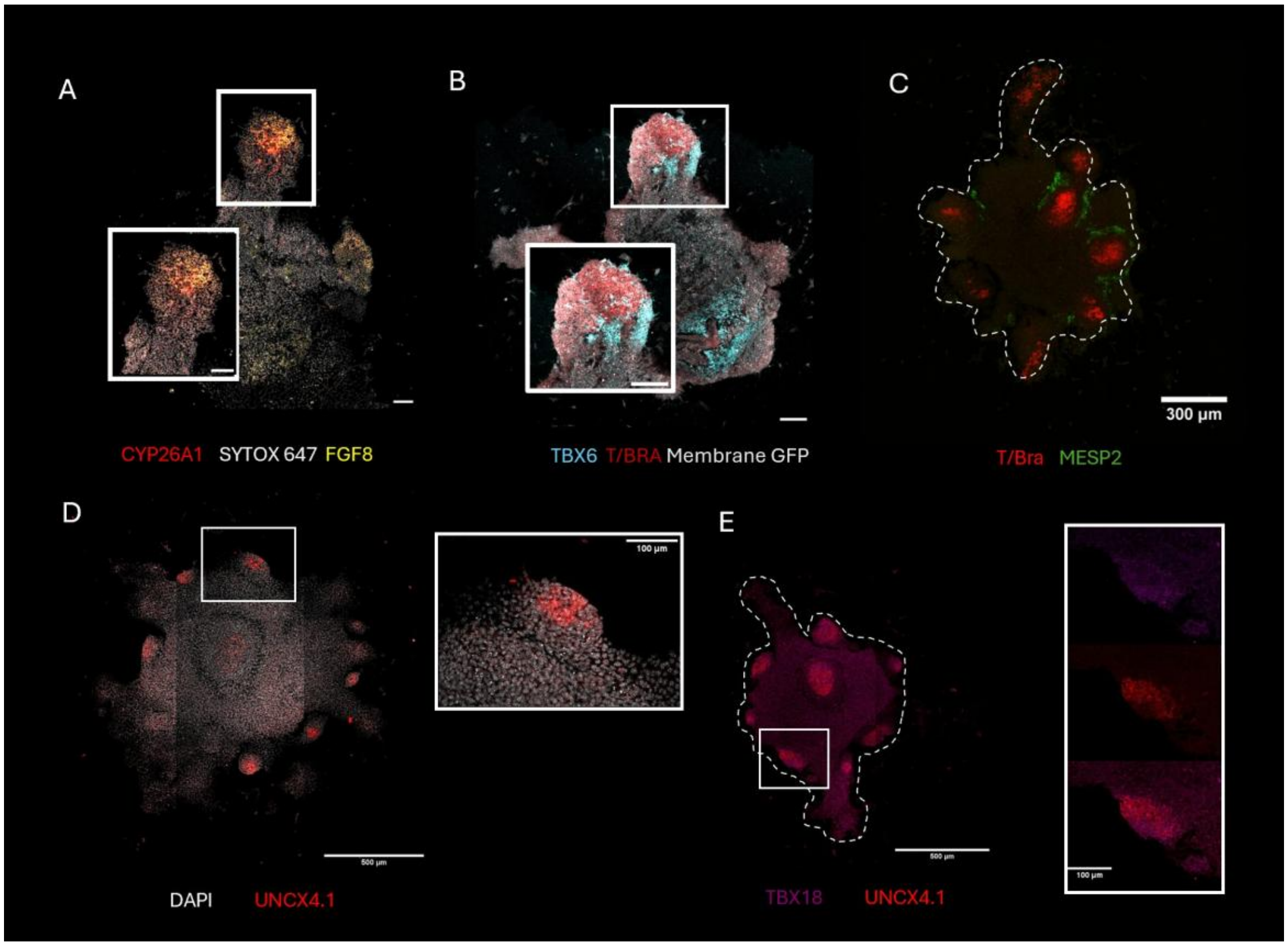


**Supplementary Fig. 7. More cell fate markers in gastruloids grown on laminin.** A) HCR staining for markers of the posterior tailbud CYP26A1 and FGF8. (FGF8, n=3, 3/3 protrusions) B) TBX6 (Presomitic mesoderm) and T/Bra (mesoderm). (TBX6, n=3, 3/3 protrusions) C) MESP2 (Presomitic mesoderm) and T/Bra (mesoderm). (MESP2, n=5) D-E) HCRs showing somitic markers UNCX4.1 and TBX18. (UNCX4.1, n=7, 26 somite clusters; TBX18, n=5, 26 somite clusters) n specifies the number of samples on laminin, and the number of protrusions or somite clusters is indicated when applicable.

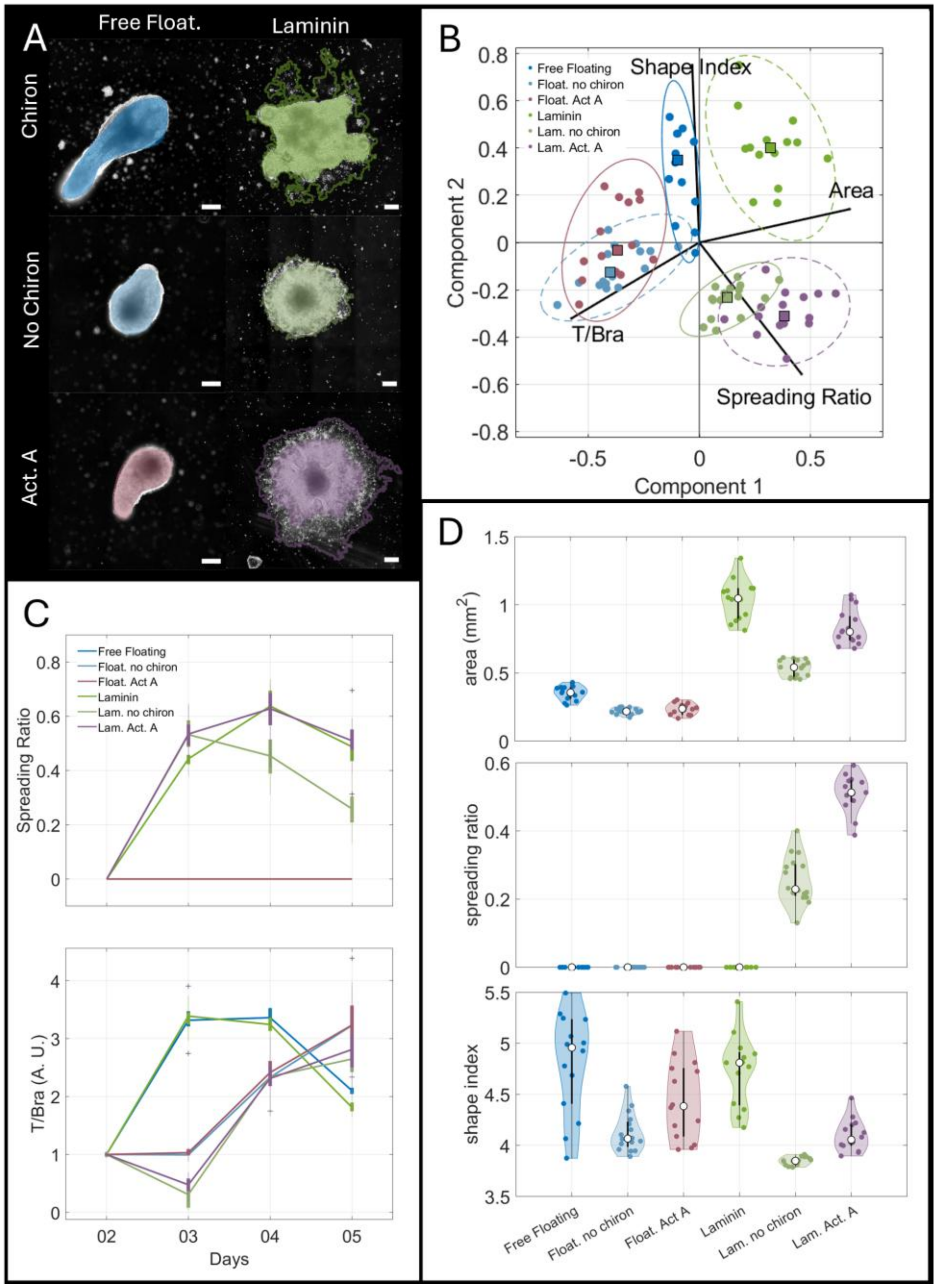


**Supplementary Fig. 8. Posterior fates are necessary for cell stream formation.** A) Gastruloid morphologies on different substrates with a Chiron pulse or an Activin A pulse on day 2. Only Chiron generates cell streams when seaded on laminin. B) Principal Component Analysis integrating different properties on day 5. C) Analysis of the spreading capability of gastruloids on different substrates (top) and T/Bra expression (bottom) from day 2 to day 5. D) Analysis of area, migratory abilities (spreading ratio) and elongation (shape index) on day 5 gastruloids grown on different coatings in the presence/absence of Chiron. See Dataset S1 for the full statistical analysis.

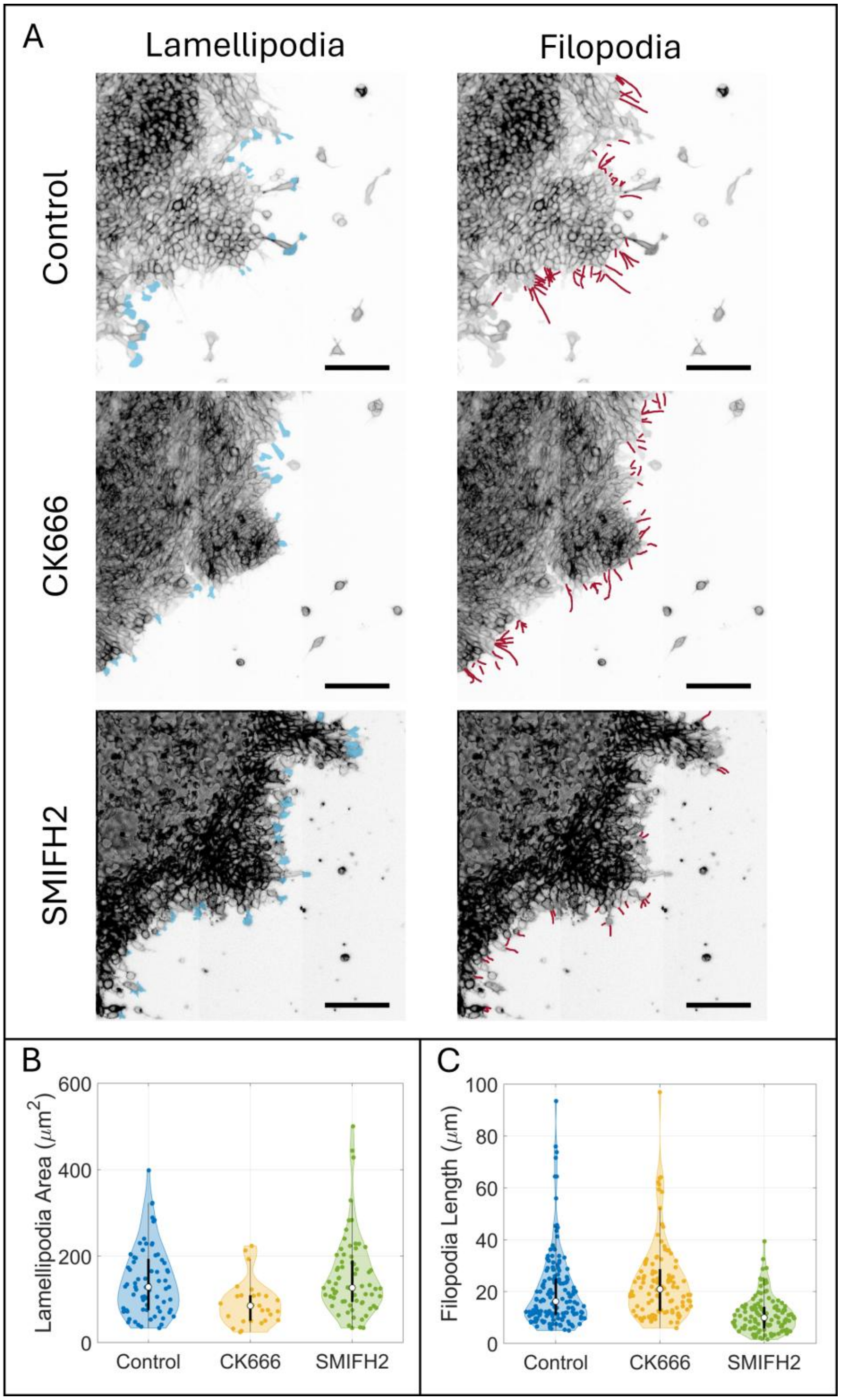


**Supplementary Fig. 9. Quantification of Lamellipodia and Filopodia on laminin gastruloids.** A) Demonstration of the manual labelling of lamellipodia (left) and filopodia (right) and its morphology with different inhibitors. B) Quantification of lamellipodia area. C) Quantification of filopodia length. See Dataset S1 for the full statistical analysis.

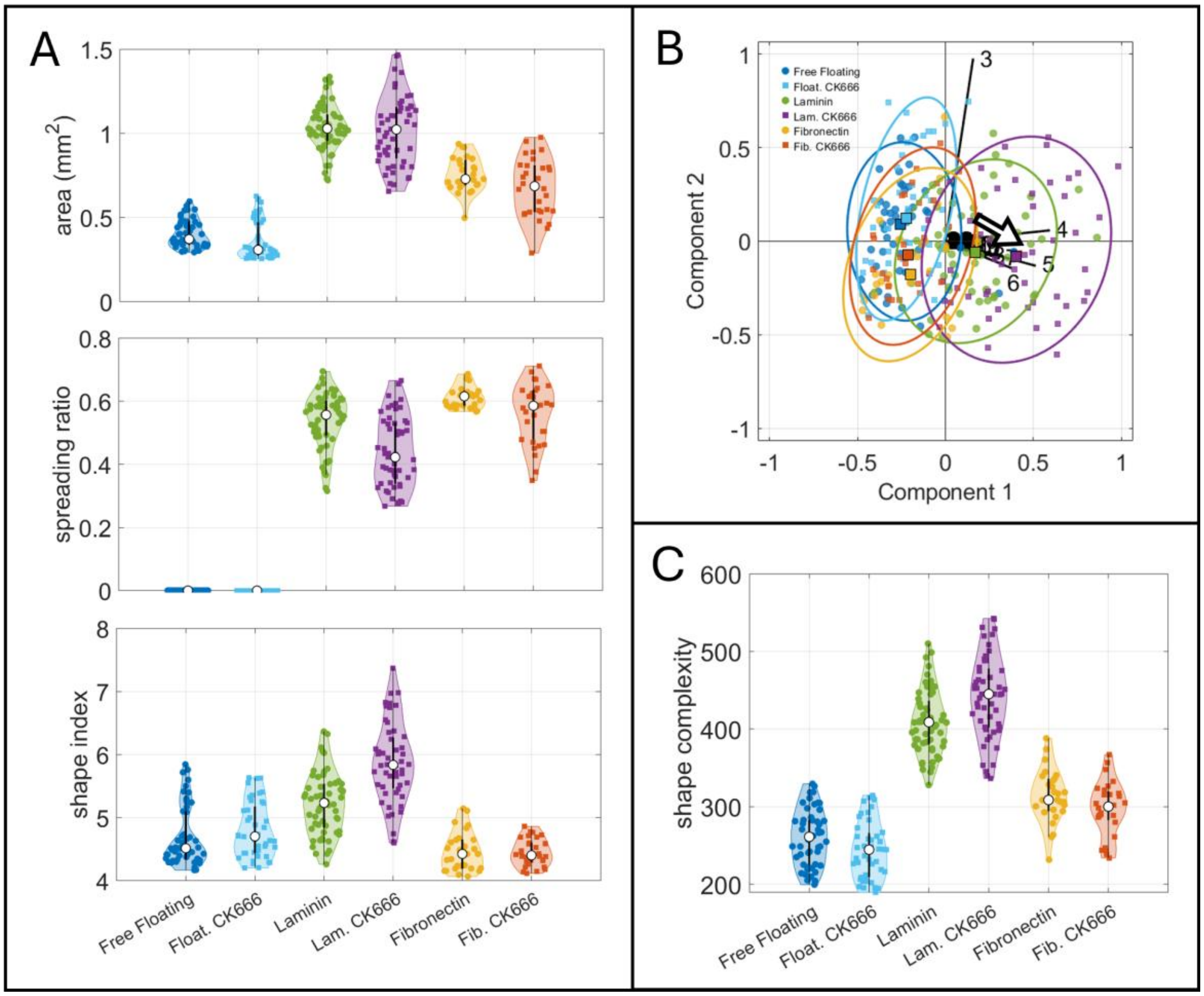


**Supplementary Fig. 10. Characterisation of lamellipodia inhibition on different substrate.** Analysis of area, migratory abilities (spreading ratio) and elongation (shape index) on day 5 gastruloids grown on different coatings in the presence/absence of CK666 50 $_{\mu M}$. B) LOCO-EFA modes for the different morphologies. Arrow indicates the need of higher frequency modes to describe the shapes on laminin gastruloids when in presence of the CK666. C) Shape complexity estimated using of the contribution of LOCO-EFA high frequency modes (see methods). See Dataset S1 for the full statistical analysis.

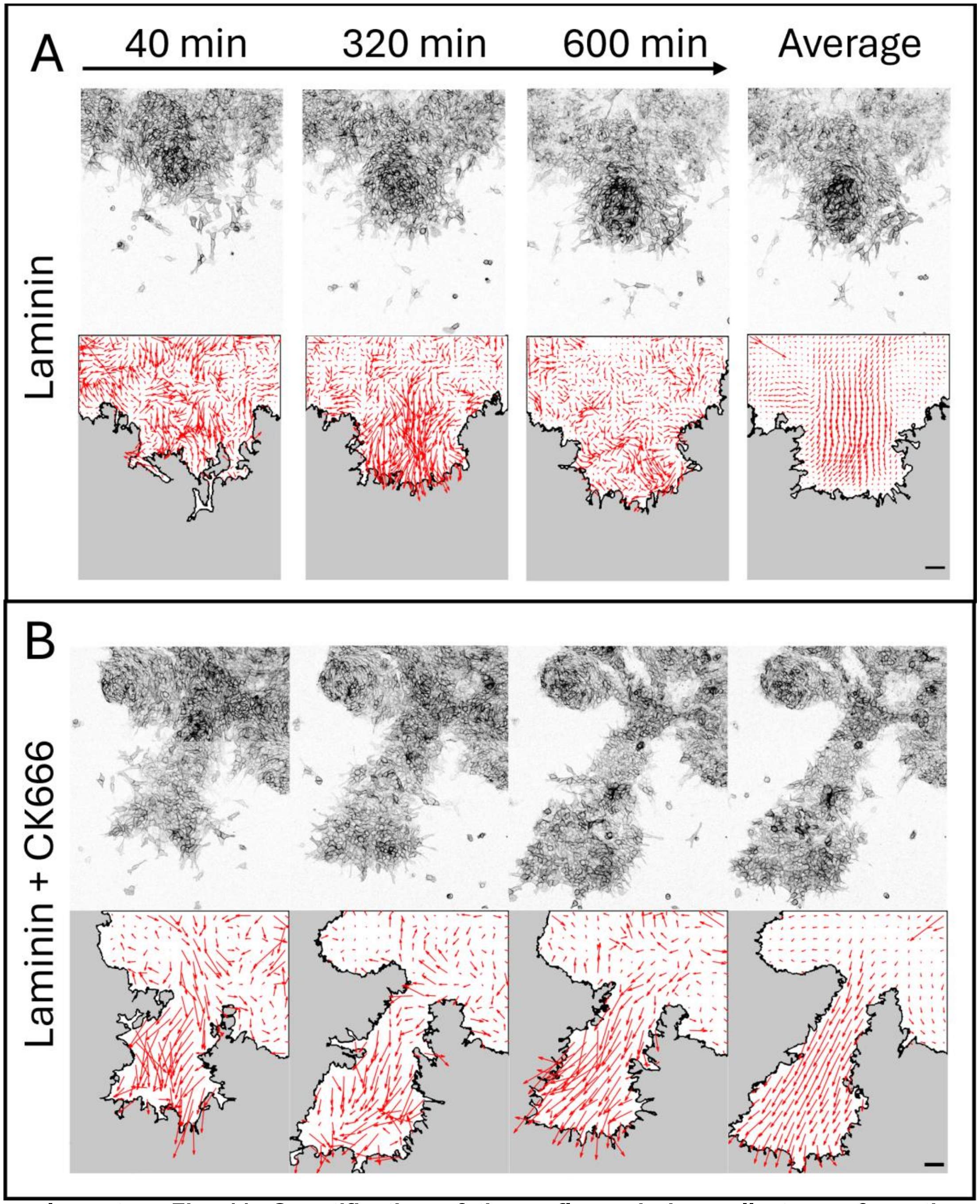


**Supplementary Fig. 11. Quantification of tissue flows during cell stream formation.** A) Particle Image Velocimetry on laminin and B) in the presence of CK666 50 $_{\mu M}$. Scale bars 200 $_{\mu m}$.

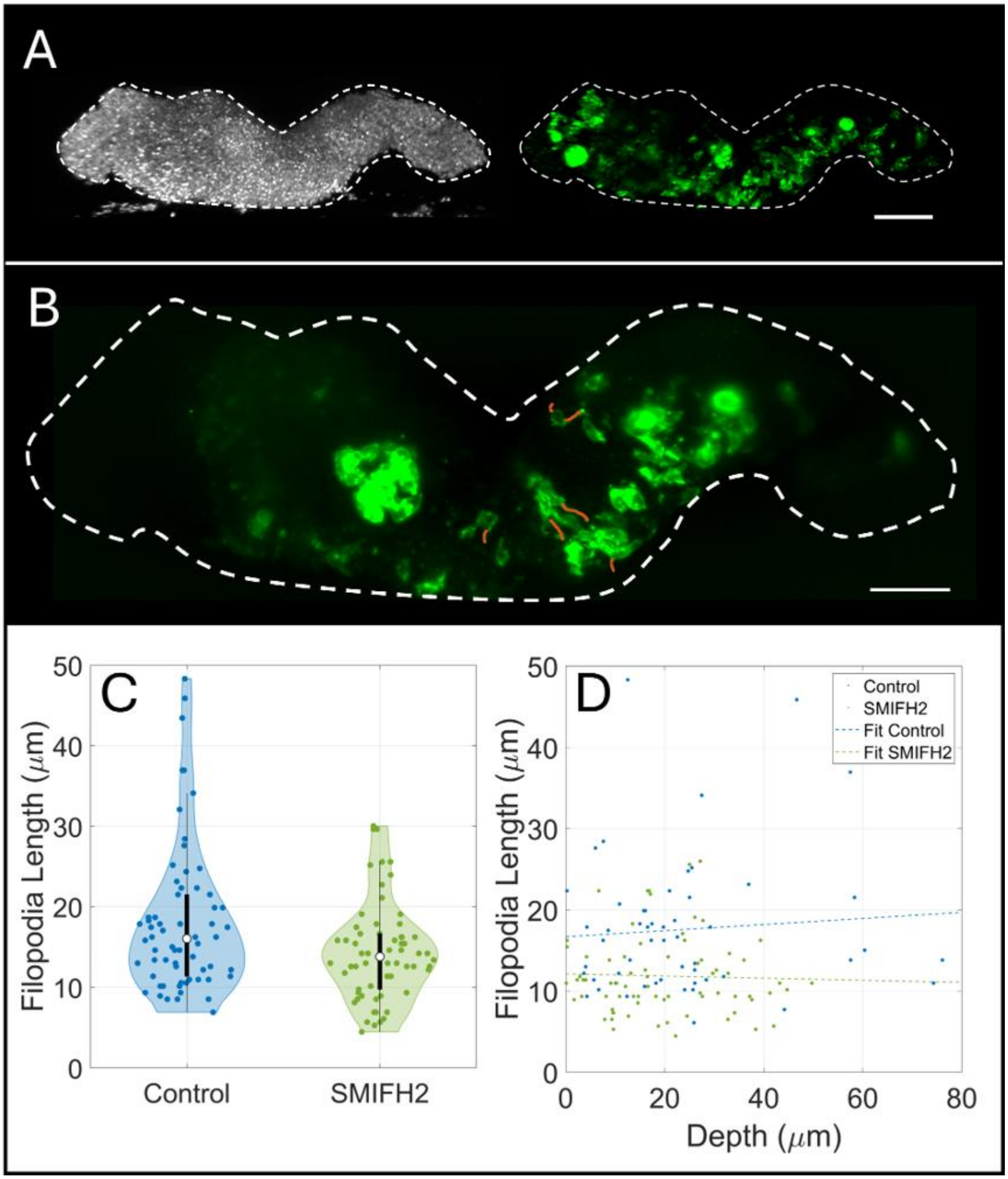


**Supplementary Fig. 12. Quantification of Filopodia on free-floating gastruloids.** A) Mosaic gastruloid (5% mGFP/ 95% SBN) for quantification of cellular protrusions. B) Demonstration of the manual labelling of filopodia. C) SMIFH2 reduces filopodia length in free-floating gastruloids. D) Filopodia length across tissue depths in control and treated gastruloids. Dotted line: least squares linear regression. See Dataset S1 for the full statistical analysis.

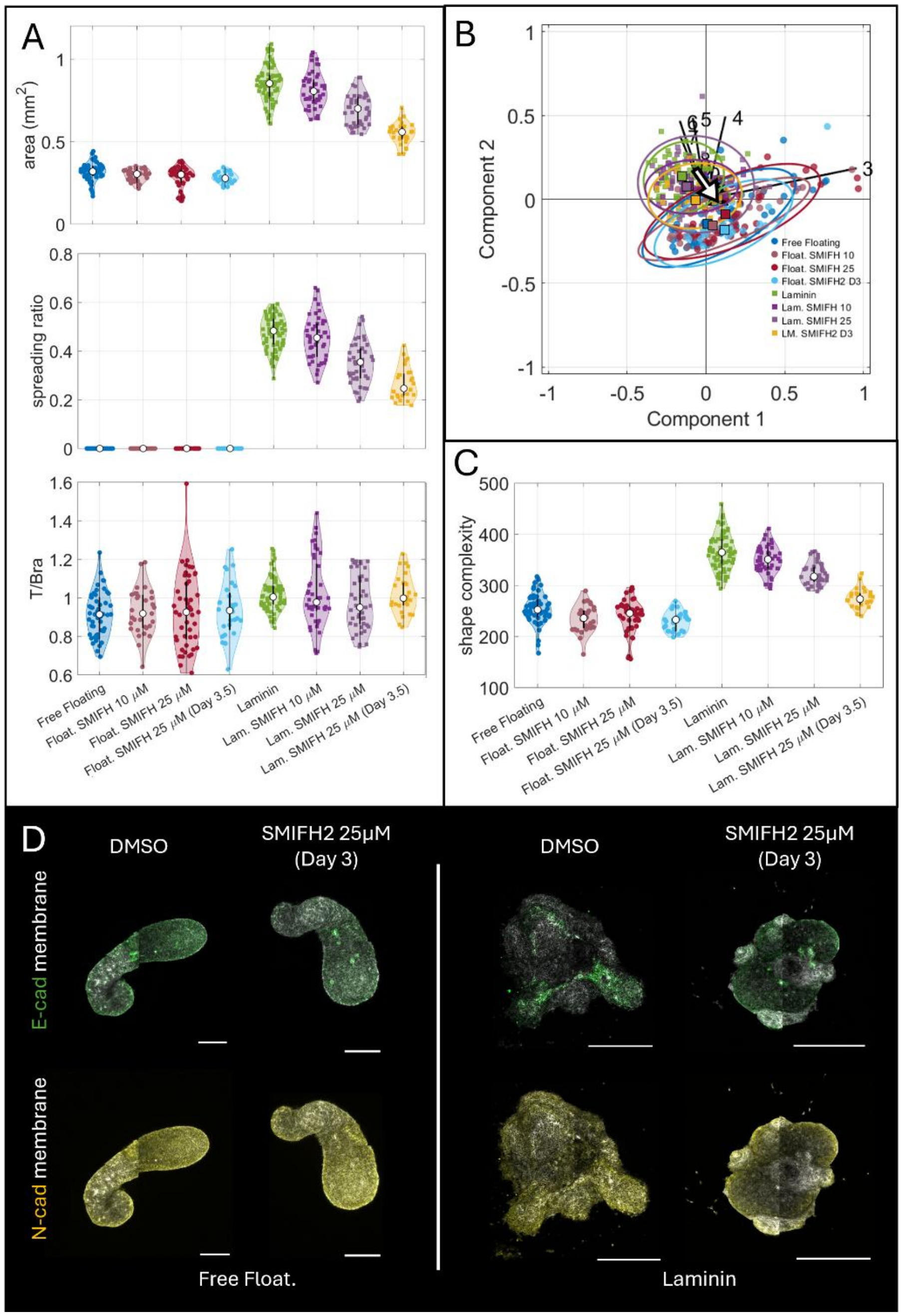


**Supplementary Fig. 13. Characterisation of filopodia inhibition on different substrates.** Analysis of area, migratory abilities (spreading ratio) and T/Bra expression on day 5 gastruloids, grown free floating or on laminin in the presence/absence of SMIFH2 25 $_{\mu M}$. B) LOCO-EFA modes for the different morphologies. Arrow indicates the decrease of higher frequency modes to describe the shapes on laminin gastruloids when in presence of the SMIFH2. C) Shape complexity estimated using of the contribution of LOCO-EFA high frequency modes (see methods). D) E-cadh and N-cadh expression on day 5 after in the presence of SMIFH2. Note: a fault in the automated stage during acquisition produced a small misalignment at the stitching seams; no image data were modified or removed. See Dataset S1 for the full statistical analysis.

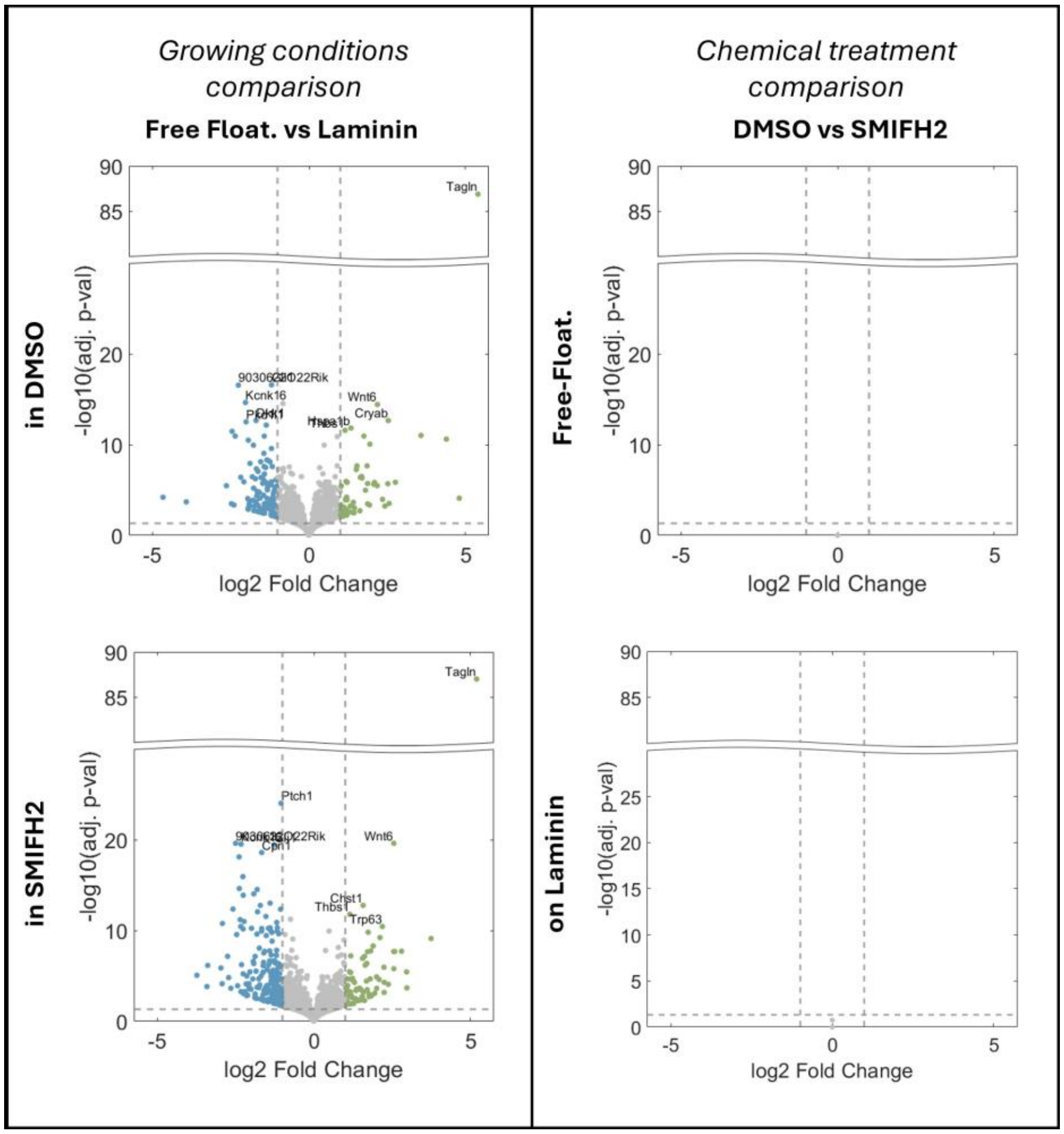


**Supplementary Fig. 14. SMIFH2 treatment does not induce transcriptional changes.** Volcano plots comparing gene expression across conditions. Left column: comparison between free-floating and laminin gastruloids, either in DMSO (top) or SMIFH2 (bottom). Both conditions show similar patterns of differential gene expression, with genes downregulated on laminin (blue) and upregulated on laminin (green). Right column: comparison between DMSO and SMIFH2 treatment, either in free-floating (top) or laminin (bottom) conditions. No differentially expressed genes are detected in either comparison, confirming that SMIFH2 blocks axis elongation through mechanical effects rather than transcriptional changes. Dashed lines indicate log2FC = ±1 and p-adj = 0.05. Selected genes are labelled.

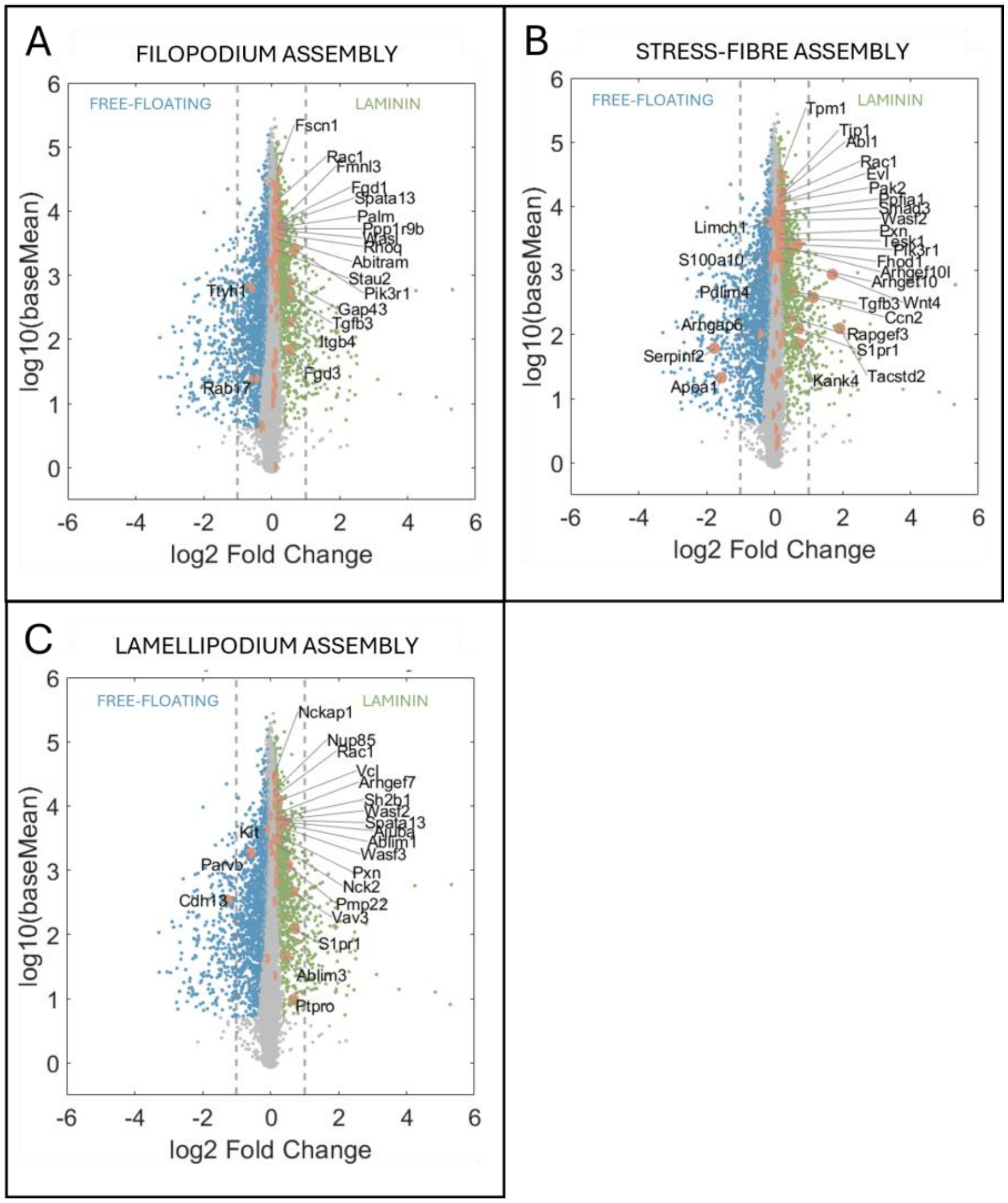


**Supplementary Fig. 15. Laminin induces regulators of filopodia, stress-fibres and lamellipodium.** MA plots highlighting expression levels of specific gene categories comparing laminin versus free-floating gastruloids. A) Regulators of filopodium assembly. B) Regulators of stress fibre assembly. s C) Regulators of lamellipodum assembly. Coloured points indicate genes with p-adj < 0.05; orange points highlight genes of interest within each category. Dashed lines indicate log2FC = ±1.

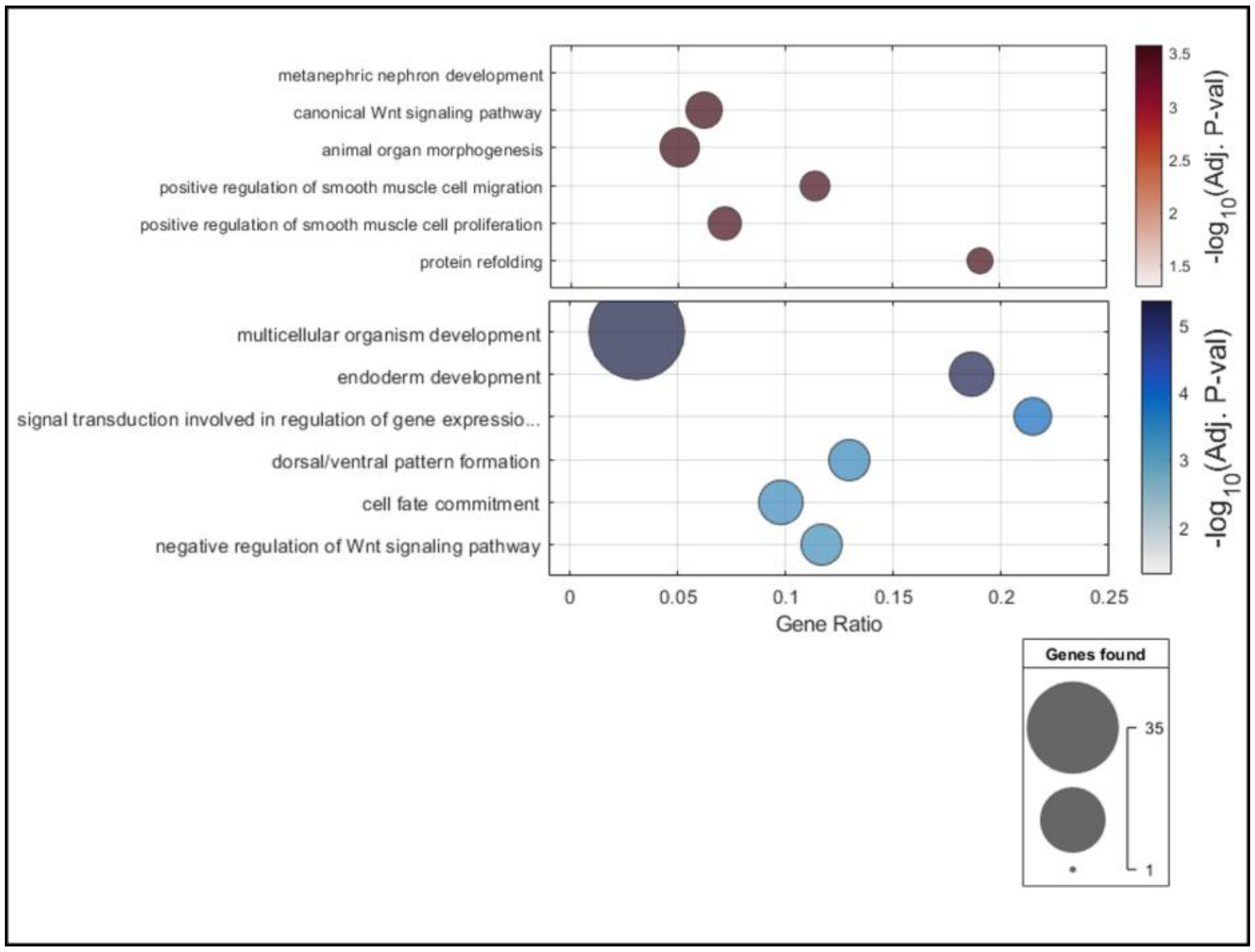


**Supplementary Fig. 16. Gene Ontology analysis of differentially expressed genes between laminin and free-floating gastruloids.** GO Biological Process enrichment analysis of genes upregulated (top, red) or downregulated (bottom, blue) on laminin compared to free-floating conditions. The x-axis shows the gene ratio (proportion of differentially expressed genes within each GO term), dot size indicates the number of genes found, and colour intensity indicates statistical significance (-log10 adjusted p-value). Only genes with |log2FC| > 1 and p-adj < 0.05 were included in the analysis.

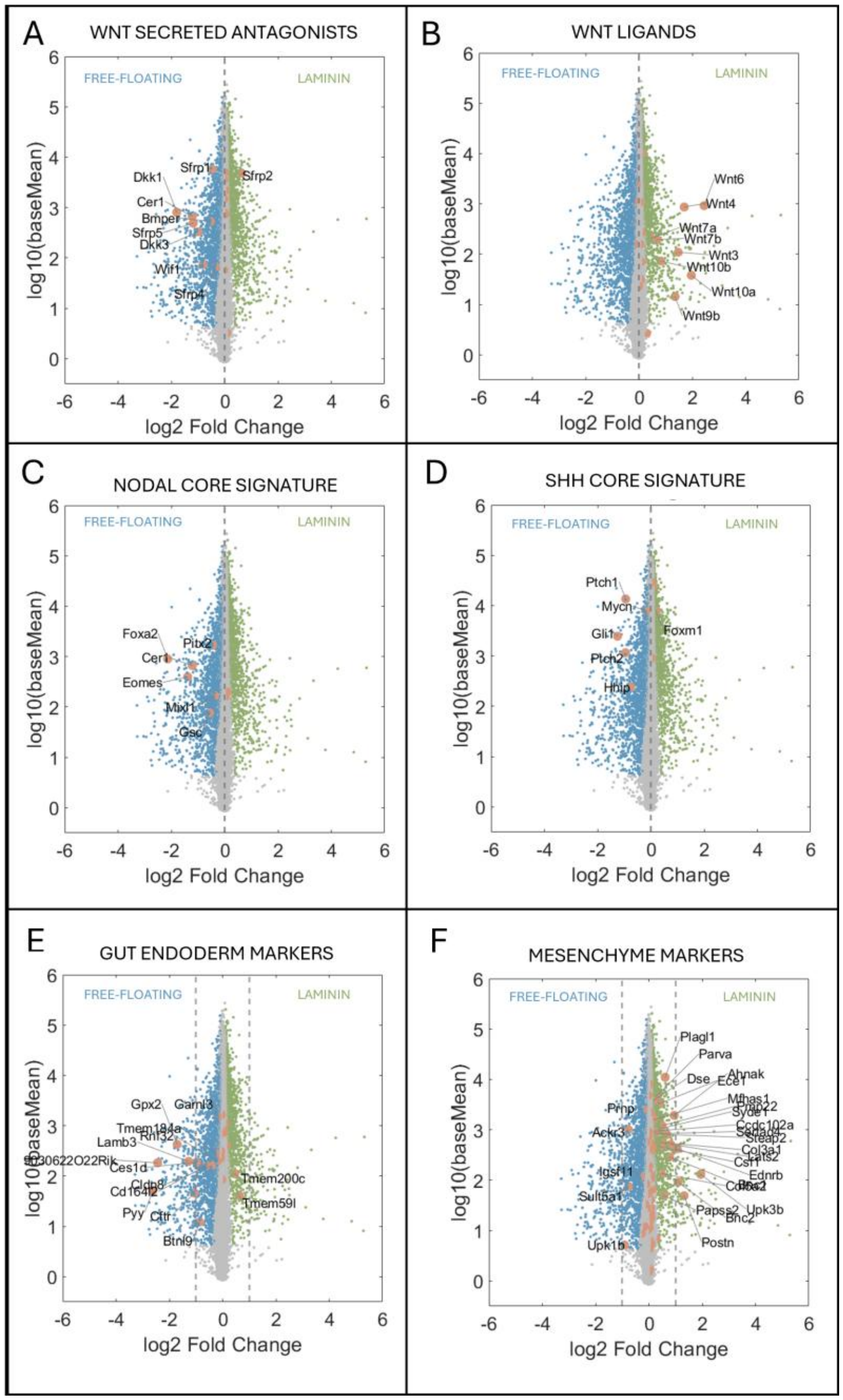


**Supplementary Fig. 17. Laminin promotes posterior migratory fates and reduces anterior primitive streak derivatives.** MA plots highlighting expression levels of specific gene categories comparing laminin versus free-floating gastruloids. A) Wnt secreted antagonists are downregulated on laminin. B) Wnt ligands are upregulated on laminin. C) Nodal pathway core signature genes are downregulated on laminin. D) Shh pathway targets are downregulated on laminin. E) Gut endoderm markers are downregulated on laminin. F) Mesenchyme markers are upregulated on laminin. Together, these data indicate that laminin biases gastruloid differentiation toward posterior, migratory fates while reducing anterior primitive streak derivatives including definitive endoderm. Coloured points indicate genes with p-adj $< 0.05$; orange points highlight genes of interest within each category. Dashed lines indicate log2FC = ±1.

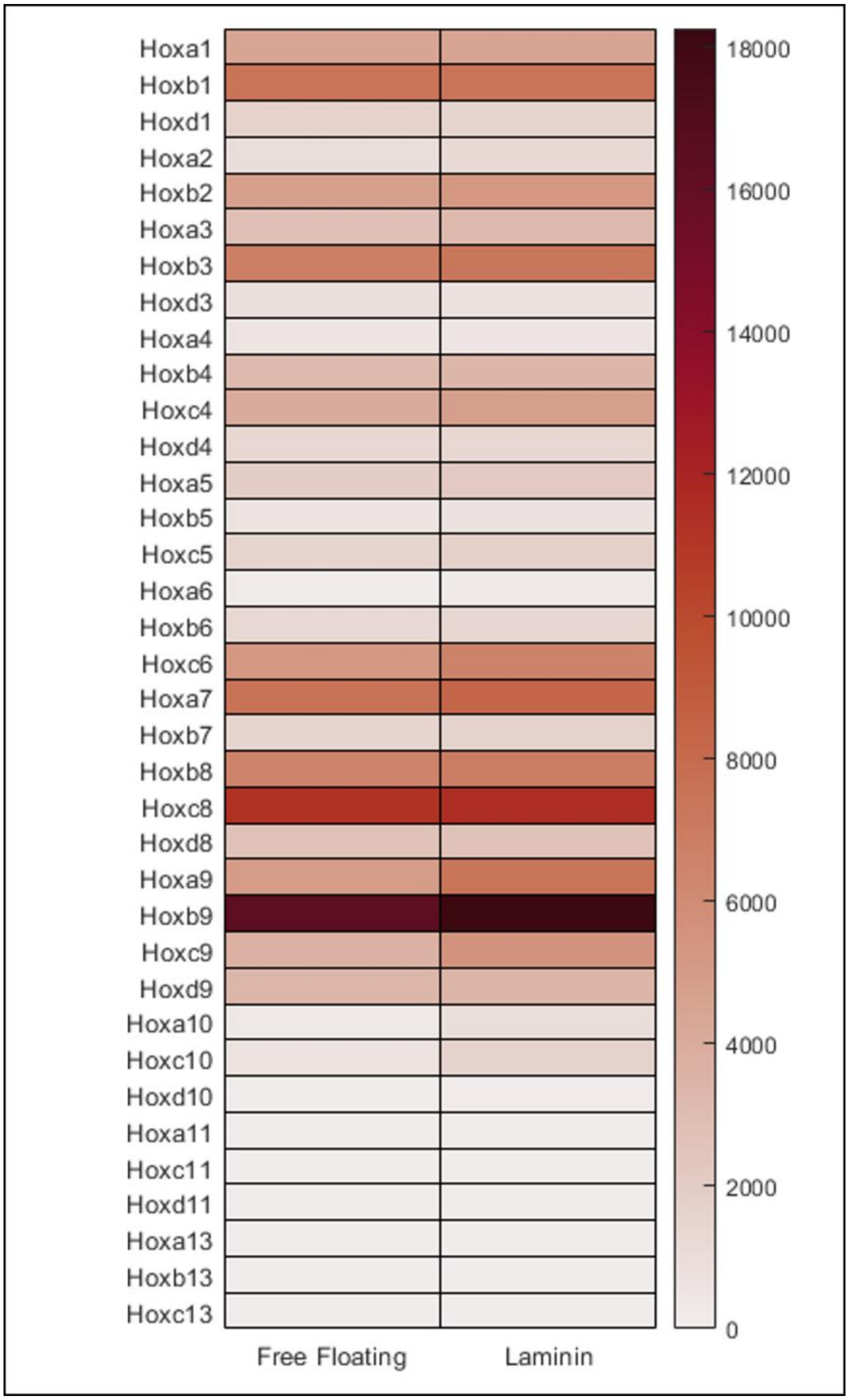


**Supplementary Fig. 18. Hox gene expression is comparable between free-floating and laminin gastruloids.** Heatmap shows mean DESeq2-normalised counts for each condition (n = 6 free-floating, n = 6 laminin). 37 of 39 Hox genes passed independent filtering and are displayed; Hoxc12 and Hoxd12 were below the expression threshold. Both conditions show similar expression patterns, with high expression of central and trunk Hox genes (Hox6–9) and low expression of caudal Hox genes (Hox10–13). This indicates both conditions retain a comparable Hox expression profile, consistent with equivalent axial identity.

**Antibody list**

| | | |
|---|---|---|
| Brachyury | 81694S | Cell Signaling Technology |
| SNAI1 | 3879 | Cell Signaling Technology |
| ZO-1 | 33-9100 | Invitrogen |
| 2P-MLC | 3674S | Cell Signaling Technology |
| N-cadherin | 7939 | Santa Cruz |
| E-cadherin | 610181 | BD Biosciences |
